# Fruiting body-associated *Pseudomonas* contact triggers ROS-mediated perylenequinone biosynthesis in *Shiraia* mycelium culture

**DOI:** 10.1186/s40643-025-00946-w

**Published:** 2025-09-26

**Authors:** Yan Jun Ma, Xin Ping Li, Jia Hui Li, Li Ping Zheng, Jian Wen Wang

**Affiliations:** 1 College of Life Sciences, Northwest Normal University, Lanzhou, China; 2 College of Pharmaceutical Sciences, Soochow University, Suzhou, China; 3 Department of Horticultural Sciences, Soochow University, Suzhou, China

**Keywords:** *Shiraia*, *Pseudomonas*, Fruiting body, Physical contact, Reactive oxygen species, Perylenequinones

## Abstract

Perylenequinones (PQs) from *Shiraia* fruiting bodies serve as potent photosensitizers for anticancer and antimicrobial photodynamic therapy (PDT). Although these fruiting bodies harbor diverse endophytic bacteria, their interactions with the host fungus remain poorly understood. In this study, we used an in vitro confrontation bioassay to investigate the interaction between *Shiraia* sp. S9 and dominant *Pseudomonas* isolates, analyzing fungal transcriptional responses and PQ biosynthesis. Comparative assessment of co-cultures with freely suspended live *P. fulva* SB1 versus dialysis membrane-separated bacteria revealed that direct physical contact is essential for eliciting fungal PQ production, particularly extracellular secretion of hypocrellin A (HA), HC, and elsinochrome A-C. Bacterial elicitation with *P. fulva* SB1 at 400 cells/mL stimulated both intracellular PQ biosynthesis and extracellular secretion, resulting in a total PQ yield of 362.2 mg/L, a 2.4-fold increase over axenic cultures. RNA-seq analysis after 24 h of co-culture identified 646 differentially expressed genes (DEGs), with 445 upregulated and 201 downregulated, showing significant enrichment in oxidative stress defense, carbohydrate metabolism, and membrane transport functions. Bacterial contact induced reactive oxygen species (ROS) generation, specifically O_2_^·−^ and H_2_O_2_, which mediated increased membrane permeability and enhanced HA production. This was achieved through upregulation of key genes involved in central carbon metabolism, polyketide synthase (*PKS*) for PQ biosynthesis, and major facilitator superfamily (*MFS*) transporter for PQ exudation. Our work provides the first evidence that the contact-dependent ROS signaling by endophytes within fruiting bodies regulates fungal secondary metabolism, offering novel insights into bacterial-fungal interactions and establishing an effective co-culture strategy for enhanced production of bioactive PQs.

**Graphical abstract:**

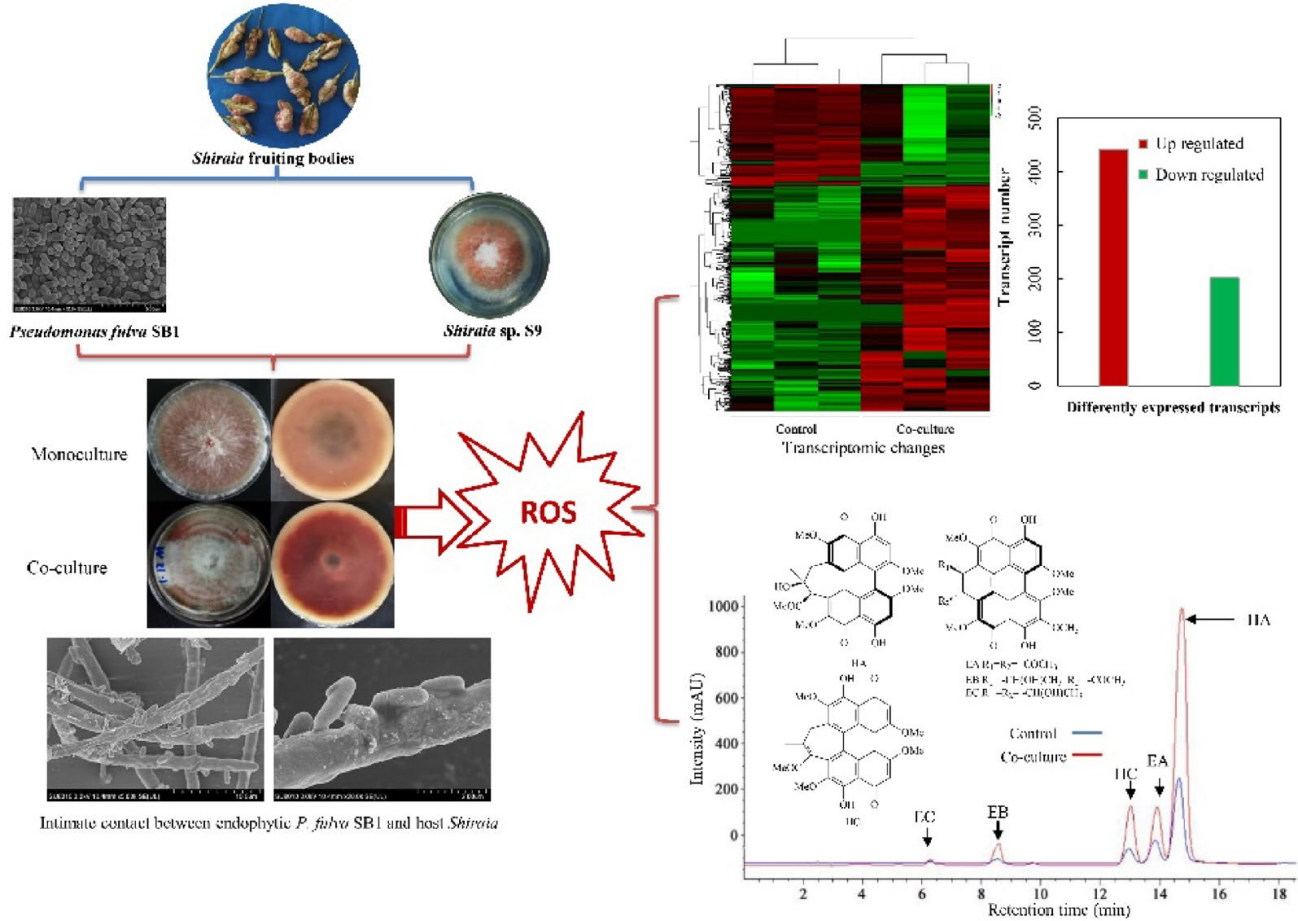

**Supplementary Information:**

The online version contains supplementary material available at 10.1186/s40643-025-00946-w.

## Introduction

The fruiting bodies of phytopathogenic *Shiraia* species, which parasitize *Brachystachyum* or *Pleioblastus* bamboos, have been used for centuries in traditional Chinese medicine to treat phlegm retention, blood stasis, and rheumatoid arthritis (Dai et al. 2017). These fungi are notable producers of perylenequinone (PQ) pigments, including hypocrellin A-D (HA-HD), shiraiachrome A-C (SA-SC) and elsinochrome A-C (EA-EC). These natural PQs share a characteristic 3,10-dihydroxy-4,9-perylenequinone chromophore, which underlies their potent photoactivity (Khiralla et al. 2022). Upon illumination, these PQs are photoactivated to transfer energy to molecular oxygen, generating reactive oxygen species (ROS) that induce oxidative damage to cellular macromolecules, ultimately triggering apoptosis in pathogenic microbes and cancer cells (Mulrooney et al. 2012). Among these compounds, hypocrellins, particularly HA and HB, have drawn considerable attention as promising photosensitizers for photodynamic therapy (PDT), owing to their high singlet oxygen quantum yields, minimal dark toxicity, and rapid systemic clearance (Estey et al. 1996). Both HA and HB exhibited tumor-selective cytotoxicity through ROS-mediated apoptotic pathways, showing potent activity against various human cancer cell lines, including ovarian cancer, epithelioid sarcoma, and lung adenocarcinoma (Zhang et al. 2024a, b). HA has also demonstrated broad-spectrum antimicrobial activity against pathogens such as *Candida albicans*, methicillin-resistant *Staphylococcus aureus*, *Pseudomonas aeruginosa*, and *Mycobacterium intracellulare*, as well as strong antileishmanial effects. In contrast, HB displayed only moderate antimicrobial activity (Ma et al. 2004). Recent findings suggest that both HA and SA can bind to the receptor-binding domain of the SARS-CoV-2 spike protein, inhibiting viral entry at nanomolar concentrations (Li et al. 2021). Additionally, EA has been shown to trigger autophagy-related cell death in melanoma cells via ROS/Atg/Parkin signaling pathways, highlighting its potential for melanoma-targeted PDT (Yao et al. 2023). Hypocrellin-based ointments have already found clinical use in treating vulvar leukoplakia and lichen amyloidosis with notable efficacy and safety profiles (Fiegler-Rudol et al. 2025). Beyond medical applications, hypocrellins have also shown promise as natural edible colorants and food preservatives, owing to their strong antimicrobial activity and dyeing properties (Su et al. 2009). Despite the promising pharmacological potential of fungal PQs, their widespread use is hindered by the difficulty of artificial cultivation of *Shiraia* fruiting bodies and the complexity of chemical synthesis (O’Brien et al. 2010). Consequently, wild-harvested *Shiraia* fruiting bodies from bamboo forests remain the primary source for PQ extraction (Li et al. 2025).

Recent studies have revealed that fruiting bodies of many macrofungi harbor diverse communities of non-pathogenic microorganisms, endophytes or associated microbes, that co-develop with the fungal host (Gohar et al. 2022). For example, *Tuber borchii* truffles exhibited rich bacterial colonization up to 10^8^ cells per gram of dry weight (DW) (Barbieri et al. 2007), while high-throughput sequencing (HTS) has uncovered 1,476 bacterial operational taxonomic units (OTUs) from *Sanghuangporus* fruiting bodies (Ma et al. 2022). The endophytic bacterial community of *Laccaria* fruiting bodies included over 16,000 amplicon sequence variants, with 23 core taxa (Zhang et al. 2024a, b). Our previous study used both culture-dependent and sequencing approaches to characterize the bacterial microbiome of *Shiraia* fruiting bodies, identifying 31 cultivable strains primarily belonging to the genera *Bacillus* (45%) and *Pseudomonas* (20%). HTS further revealed 723 OTUs spanning 30 phyla and 364 genera, with *Bacillus* (10.86%) and *Pseudomonas* (4.37%) as dominant taxa (Ma et al. 2019a). While the diversity of endophytic bacteria in fungal fruiting bodies is well documented, their ecological roles and physiological impacts on the fungal host remain poorly understood. For instance, *Rhizopogon luteolus* fruiting body-associated proteobacteria were shown to enhance fungal hyphal growth (Ayudhya et al. 2019). Similarly, *Dietzia aurantiaca* promoted growth of *Tricholoma matsutake*, whereas *Mycetocola lacteus* exerted inhibitory effects (OH et al. 2018). In *Agaricus bisporus* fruiting bodies, endophytic *Pseudomonas* strains modulated ethylene production, promoting both hyphal growth and primordia formation (Chen et al. 2013). The substrate preparation, nutrient provision, growth stimulation, and pathogen protection have been included in the positive roles of bacteria throughout the life cycle of *A. bisporus* (Braat et al. 2022). Additionally, *T. borchii*-associated proteobacteria contributed to the formation of key aroma compounds by converting methionine into thiophene derivatives (Splivallor et al. 2015). In our previous work, we demonstrated that co-culturing *Shiraia* sp. S9 with its endophytic bacterium *P. fulva* SB1 significantly enhanced fungal PQ production (Ma et al. 2019b). While recent studies have demonstrated volatile-mediated induction of hypocrellins in *Shiraia* by endophytic *P. fulva* SB1 (Xu et al. 2023) and *Rhodococcus* sp. No. 3 (Zheng et al. 2025), the role of direct physical contact remains unexplored. Herein, we revealed a novel contact-dependent mechanism whereby the endophytic bacterium *P. fulva* SB1 triggered a rapid ROS burst in *Shiraia* sp. S9, which in turn functioned as a central signaling molecule to orchestrate extensive metabolic rewiring and regulated membrane permeabilization. This cascade culminated in the enhanced biosynthesis and secretion of a broad spectrum of PQ pigments. This study provides the first evidence for contact-mediated ROS signaling as a key modulator of fungal secondary metabolism within fruiting bodies, offering a new paradigm for understanding intimate bacterial-fungal interactions.

## Materials and methods

### Microbial strains and cultivation

The host fungus *Shiraia* sp. S9 and its endophytic bacteria were isolated from fresh *Shiraia* fruiting bodies collected from *Brachystachyum densiflorum* bamboo shoots, as described in our previous study (Ma et al. 2019a). The fungal strain *Shiraia* sp. S9 and the bioactive endophytic *P. fulva* SB1 have been deposited in China General Microbiological Culture Collection Center (CGMCC) under accession numbers CGMCC 16,369 and CGMCC19391, respectively. *Shiraia* sp. S9 was routinely maintained on potato dextrose agar (PDA) plates at 4 °C, while *P. fulva* SB1 was preserved in Luria-Bertani (LB) agar plate under the same condition. For PQ production, mycelium cultures of *Shiraia* sp. S9 were grown in 150-mL Erlenmeyer flasks containing 50 mL production medium, incubated at 28 °C with shaking at 150 rpm for 8 days. The medium composition and culture conditions followed those reported in our previous study (Wang et al. 2024).

## Confrontation assay and bacterial-fungal co-culture

The interactive effects between *Shiraia* sp. S9 and *Pseudomonas* isolates were evaluated using a confrontation assay as described by Ma et al. (2019b). A 5-mm mycelial plug was inoculated at the center of a 90-mm PDA Petri dish and incubated at 28 °C in the dark for 4 days. Then, 10 µL aliquots of bacterial suspension (OD_600_ = 0.8) were streaked in parallel lines approximately 70 mm apart using a sterile inoculation loop. Control plates received sterile LB broth instead of bacteria. Plates were further incubated for 4 additional days before analysis.

For liquid mycelium culture experiments, an optimized two-stage system was used (Ma et al. 2019b). Mid-exponential phase bacterial cultures (OD_600_ = 0.8) were harvested by centrifugation and resuspended in sterile distilled water. A final concentration of 400 cells/mL of bacterial suspension was introduced into 6-day-old fungal cultures (50 mL) in 150-mL flasks. To simulate non-contact interaction (Mearns-Spragg et al. 1998), bacterial cells were enclosed in a sterile dialysis bag (molecular weight cut-off: 8–14 kDa, 1 × 5 cm^2^; Sinopharm, Nanjing, China). Specifically, 2 mL of *P. fulva* SB1 suspension (5 × 10^3^ cells/mL) was sealed inside the bag, which was then immersed into the fungal culture medium.

## Determination of PQ production, medium pH and residual glucose

PQs from agar plates, mycelia and broth were extracted according to Liu et al. (2018). Quantification was performed using high-performance liquid chromatography (HPLC) method reported by Tong et al. (2017). The individual PQ were qualified using a reverse-phase Agilent 1260 Infinity II system (Agilent, Co., Wilmington, USA) equipped with a HC-C18 column (250 × 4.6 mm). Elution was carried out using mobile phase acetonitrile: water (65: 35, v/v) at 1 mL/min and at 465 nm. Total production of HA refers to the sum of intracellular and extracellular HA. Residual glucose in the culture broth was measured using the anthrone-sulfuric acid method (Ebell 1969). Broth pH value was monitored using a calibrated FE20 pH meter (Mettler Toledo, Greifensee, Switzerland).

## RNA extraction, library preparation and transcriptome sequencing

Fungal hyphae were harvested 24 h after co-culture initiation (day 6 of fungal growth). Three biological replicates were collected respectively for monoculture controls and co-culture treatments. Total RNA was extracted using the mirVana^TM^ RNA isolation Kit (Ambion-ABI, Austin, USA), and RNA quality was assessed using the RNA Nano 6000 Assay Kit on the Agilent Bioanalyzer 2100 (Agilent Technologies, Santa Clara, USA). cDNA library preparation and Illumina sequencing were performed using the HiSeq^TM^2000 platform (Illumina, San Diego, USA), as described previously (Lei et al. 2017). Six RNA-seq libraries were generated and deposited in the NCBI Sequence Read Archive (SRA) under accession Numbers SRR7694664-SRR7694669.

Gene functional annotation was conducted using BLAST (e < 1^e − 5^) against the NCBI NR, Swiss-Prot, and KOG/COG databases (Altschul et al. 1990; Kanehisa et al. 2008). GO terms were assigned via Swiss-Prot ID mapping, and KEGG pathway annotation was conducted using the KAAS server. Reads were aligned to the reference unigene set using Bowtie2 (v2.4.2), and transcript abundance was quantified with eXpress (v1.5.1) to generate FPKM values and raw counts (Trapnell et al. 2010; Langmead et al. 2012). Differentially expressed genes (DEGs) were identified using DESeq2 with the nbinomTest function, applying a dual threshold: fold change (|FC| ≥ 1.5 and false discovery rate (FDR)-adjusted *p*-value < 0.05) (Roberts and Pachter 2013).

## Quantitative real-time PCR (qRT-PCR) validation

Total RNA was extracted using RNAprep pure Plant Kit (Tiangen, Beijing, China). Gene-specific primers, including those for the reference gene 18 S rRNA, are listed in Table S1. qRT-PCR was performed as described previously (Ma et al. 2018), and the relative gene expression was calculated from the efficiency-corrected ΔΔCt method (Livak and Schmittgen 2001).

### ROS detection and antioxidant enzyme assays

Intracellular ROS levels were assessed using 2’,7’-dichlorodihydrofluorescein diacetate (DCFH-DA, Beyotime Biotechnology, Haimen, China), as described by You et al. 2013. Fluorescence imaging was conducted with an Olympus CKX41 inverted microscope equipped with a FITC filter. Superoxide anion (O₂^·−^) and hydrogen peroxide (H_2_O_2_) levels were determined according to Pan et al. (2014). Enzymatic responses to oxidative stress were measured in mycelial extracts prepared on ice. Activities of NADPH oxidase (NOX), superoxide dismutase (SOD), catalase (CAT), peroxidase (POD), glutathione peroxidase (GSH-Px), and reduced glutathione (GSH) content were quantified using commercial kits (Beyotime Biotechnology, Haimen, China) and normalized to protein content (U/mg protein or µmol/g fresh weight), following the protocols of Deng et al. (2013) and Tongul et al. (2014).

To evaluate the role of ROS in PQ biosynthesis, fungal cultures were pretreated for 1 h with ROS modulating agents: 1 mM H_2_O_2_ (ROS donor), 0.1 mM ascorbic acid (VC, ROS scavenger) or 5 µM diphenyleneiodonium chloride (DPI, a NOX inhibitor), based on Lu et al. 2019, prior to the bacterial addition.

## Membrane permeabilization and fatty acid composition

Cell membrane integrity was evaluated using SYTOX Green staining (Thevissen et al. 1999). Fungal hyphae were incubated with 0.5 µM SYTOX Green in the dark for 30 min, washed and observed using fluorescence microscopy (CKX41, Olympus, Tokyo, Japan). Fluorescence intensity was quantified using ImageJ 1.53. Membrane fatty acids were extracted following the method of Li et al. (2013) and analyzed using an Agilent 7890B gas chromatograph (Agilent Technologies, Santa Clara, CA, USA) equipped with a DB-23 capillary column (30 m × 0.25 mm). Fatty acids were identified by comparing retention times with authentic standard compounds.

## Scanning electron microscopy (SEM)

To visualize the spatial interaction of endophytes with the host, fresh *Shiraia* fruiting bodies were washed, sectioned into 3 × 3 × 3 (mm) pieces, and fixed overnight at 4 °C in 2.5% (v/v) glutaraldehyde. Samples were processed by dehydration, freeze-drying, and gold sputter-coating before SEM imaging using a Hitachi S-4700 microscope, following the protocol of Wang et al. (2015). For co-culture observations, samples were collected after 36 h, fixed and prepared for SEM as described above.

### Statistical analysis

All experiments were performed with three independent replicates (six plates or flasks per replicate). Data are presented as mean ± standard deviation (SD). Statistical significance was assessed using Student’s t-test for two-group comparisons and one-way ANOVA with Dunnett’s post hoc test for multiple comparisons. A *p*-value < 0.05 was considered statistically significant.

## Results

### Screening of *Pseudomonas* endophytes for PQ elicitation in co-culture

**Fig. 1:**
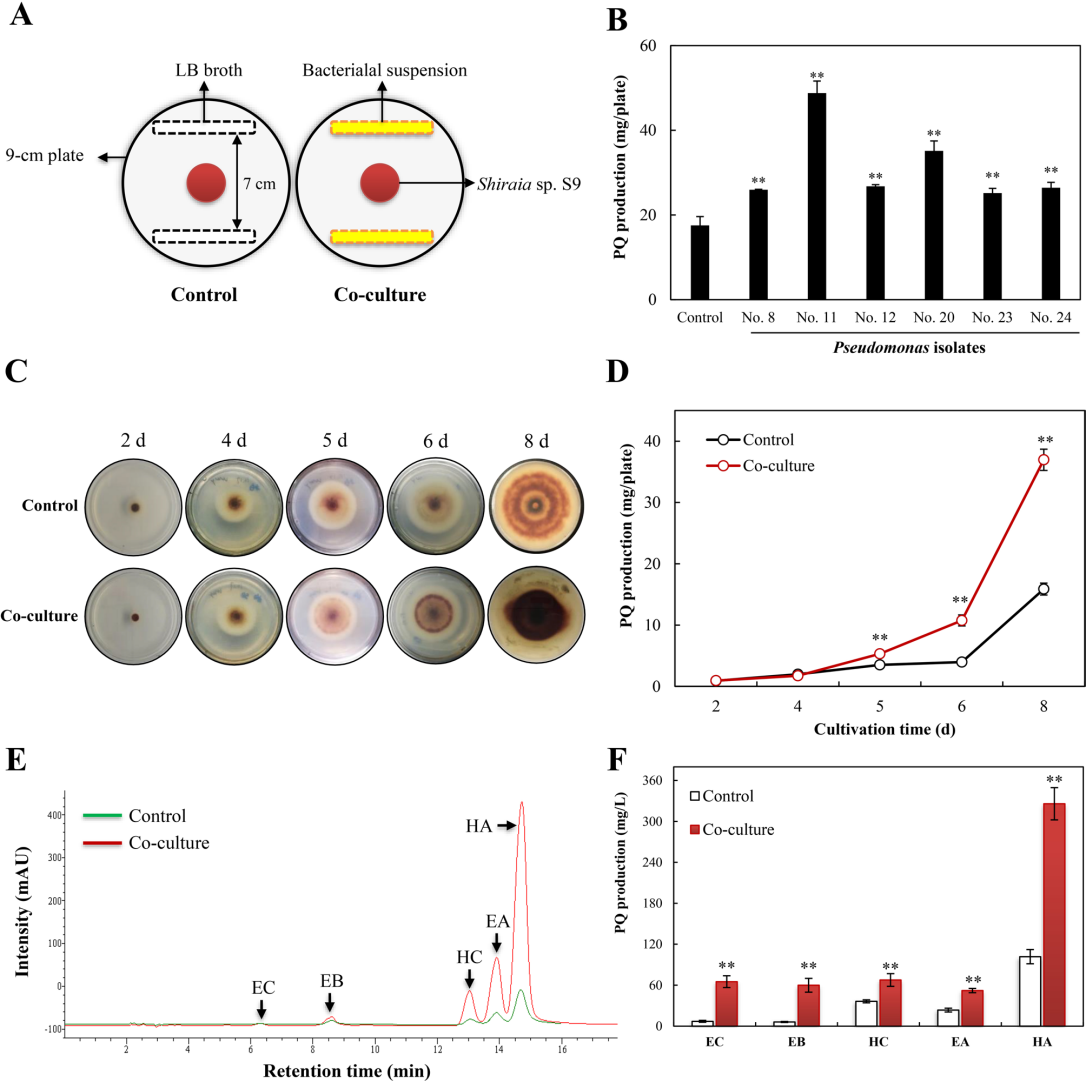
Effects of endophytic *Pseudomonas* on fungal perylenequinone (PQ) production of *Shiraia* sp. S9. (**A**) Schematic diagram of bacterial-fungal co-culture in PDA plate. (**B**) The effects of *Pseudomonas* isolates on fungal PQ production of *Shiraia* sp. S9 in solid-state culture. *Pseudomonas* isolate No. 8 (*P. putida*), No. 11 (*P. fulva* SB1), No. 20 (*P. parafulva*), No. 23 (*P. putida*), No. 24 (*P. putida*). (**C**) Colony morphology and red pigment production during the confrontation on PDA plate. *P. fulva* SB1 was streaked on day 4. (**D**) Total fungal PQ production in response to bacterial confrontation on PDA. (**E**) Chromatographic profiling of *P. fulva* SB1-induced individual PQ of *Shiraia* sp. S9 at day 8. *P. fulva* SB1 was inoculated at 400 cells/mL on day 6 of mycelium culture at 150 rpm, 28℃. (**F**) Quantification of individual PQ production of *Shiraia* sp. S9 on day 8 of the mycelium culture. The culture without SB1 treatment was used as control. **p < 0.01 versus control group.

To identify potential endophytic bacterial elicitors of PQ production, six *Pseudomonas* isolates from *Shiraia* fruiting bodies were co-cultured with *Shiraia* sp. S9 (Fig. 1A). Following 4 d of co-cultivation, the *Pseudomonas* isolates significantly enhanced fungal PQ production. Among these, *P. fulva* SB1 (No. 11) demonstrated the most pronounced stimulatory effect, elevating PQ yield to 48.8 mg/plate, a 2.8-fold increase over the control (Fig. 1B). The elicitation capacity of *P. fulva* SB1 was further verified over an extended 8 d co-culture period (Fig. 1C), during which total PQ production increased by 52–133% (Fig. 1D). In liquid mycelial cultures inoculated with *P. fulva* SB1 at 100–600 cells/mL, the highest PQ production was achieved at 400 cells/mL (Fig. S1), whereas specific PQs were differentially induced (Fig. 1E). Both EB and EC were induced from negligible levels to very high levels (59.9 mg/L and 65.2 mg/L respectively). Substantial enhancements were observed for HC (67.6 mg/L; 1.9-fold), EA (52.1 mg/L; 2.2-fold), and HA (325.9 mg/L; 3.2-fold) compared to controls.

### Comparison of PQ production in contact and non-contact co-culture systems

**Fig. 2:**
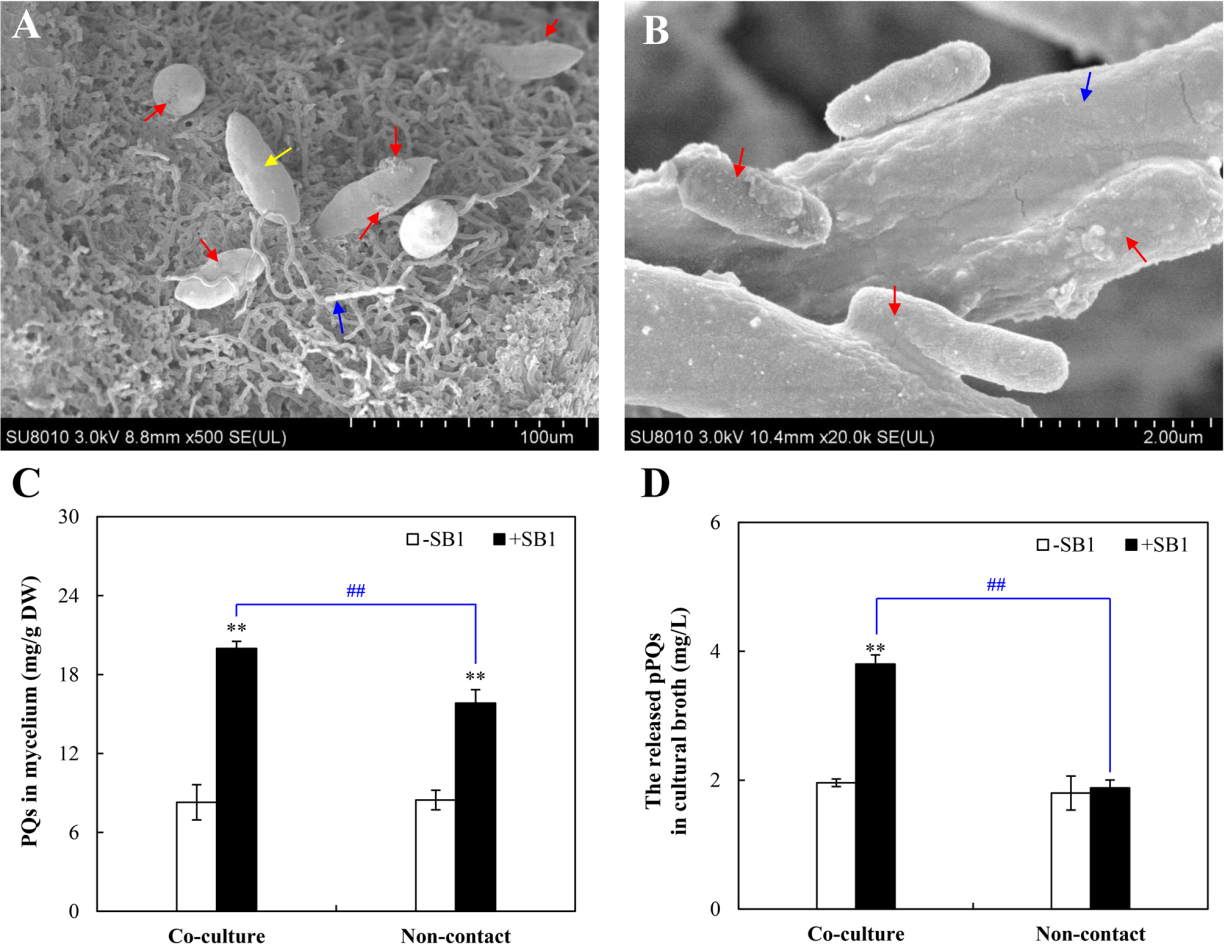
**(A)** Scanning electron micrographs (SEM) of transverse section of *Shiraia* fruiting body. Red arrow indicates bacteria. Yellow arrow indicates ascospore. Blue arrow indicates pseudoparenchyma. (**B)** SEM image of the bacterial-fungal physical attachment after 36 h co-culture of *Shiraia sp.* S9 and *Pseudomonas fulva* SB1. Red arrow indicates bacteria. Blue arrow indicates hyphae. Effects of *P. fulva* SB1 on PQ contents in mycelium (**C)** and the released PQ (**D**) in mycelium culture of *Shiraia* sp. S9 on day 8. *P. fulva* SB1 was inoculated at 400 cells/mL on day 6 of mycelium culture at 150 rpm, 28℃. **p < 0.01 versus “-SB1” group. “-SB1” group refers control group without the bacteria. ^##^*p* < 0.01 denotes the significant difference between the “+SB” in fungal co-culture and non-contact co-culture with bacteria

SEM observation revealed extensive colonization of endophytic bacteria within the asci and on mature ascospore surfaces of *Shiraia* fruiting bodies (Fig. 2A). In co-culture, intimate physical contact was observed between *P. fulva* SB1 and *Shiraia* sp. S9 hyphae, with bacterial cells densely adhered to fungal surfaces and embedded deeply within hyphae (Fig. 2B). To delineate the role of physical interaction in PQ elicitation, *Shiraia* sp. S9 was co-cultured with live *P. fulva* SB1 or exposed to the bacterium under non-contact conditions (separated by dialysis membranes). While fungal biomass remained statistically unchanged across treatments (Fig. S2A), live *P. fulva* SB1 significantly stimulated both intracellular PQ biosynthesis by 141% (co-culture group in Fig. 2C) and extracellular PQ by 94% (co-culture group in Fig. 2D), culminating in a total PQ yield of 362.2 mg/L (Fig. S2B), representing a 2.4-fold enhancement over axenic control. In contrast, non-contact exposure solely elevated intracellular PQ accumulation without enhancing PQ secretion (Non-contact group in Fig. 2C, D). The stark contrast between the contact and non-contact systems, where the latter permits the exchange of diffusible metabolites but prevents physical interaction, demonstrated that while bacterial small molecules may contribute marginally to intracellular PQ accumulation, direct physical contact was the indispensable trigger for the full elicitation response, particularly the robust secretion of PQs into the extracellular environment.

### Transcriptomic changes in response to the bacterial elicitation

**Fig. 3:**
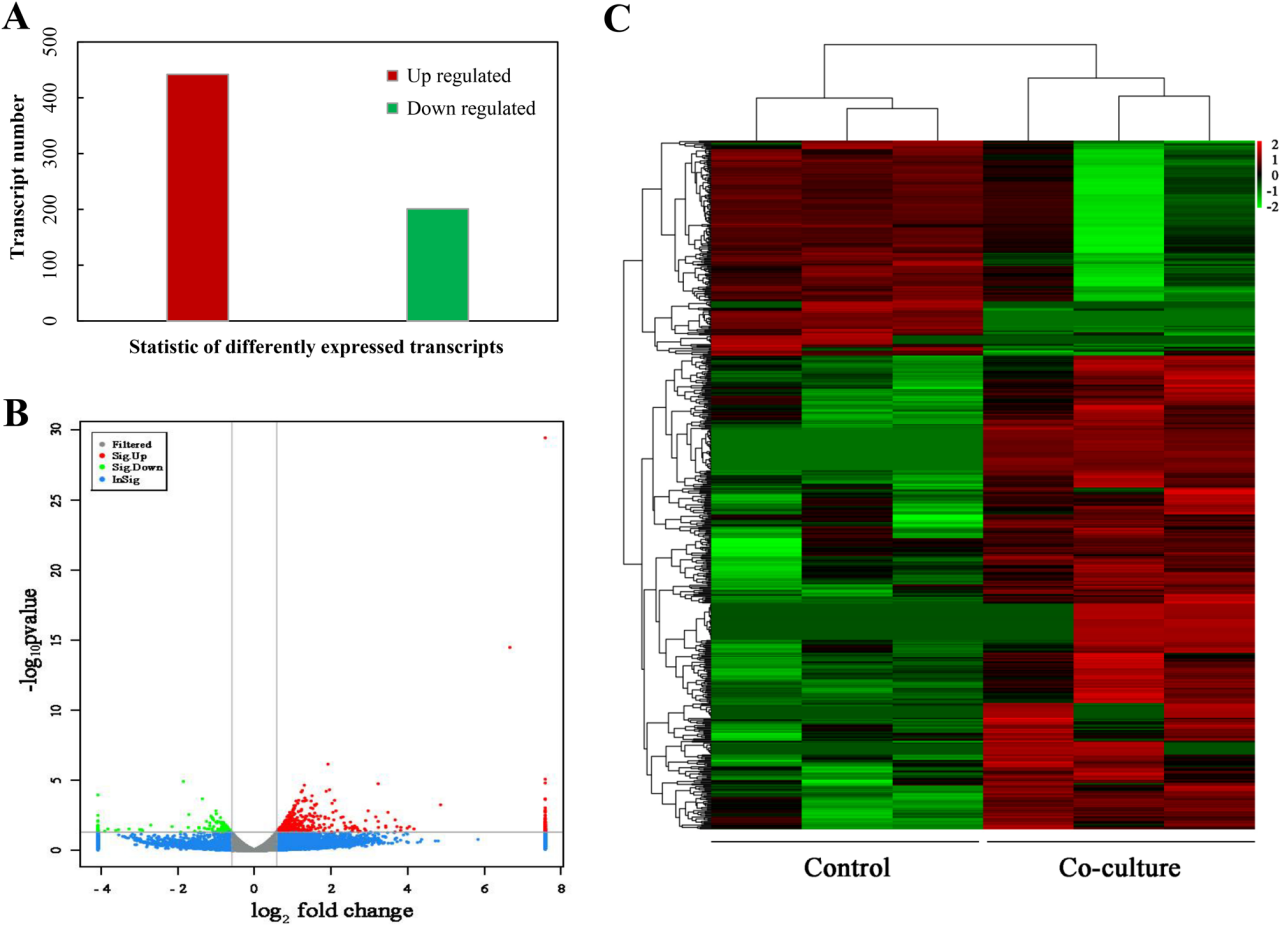
Transcriptomic changes of *Shiraia* sp. S9 co-cultured with *Pseudomonas fulva* SB1 after 24 h. (**A)** Differentially expressed genes between control group and co-culture group. Bar plot shows counts of upregulated (red) and downregulated (green) genes (*p* < 0.05, |FC| ≥ 1.5). *P. fulva* SB1 was inoculated at 400 cells/mL on day 6 of mycelium culture of *Shiraia* sp. S9 at 150 rpm, 28℃. The mycelial samples were taken after 24 h of the co-culture. Three independent biological replicates were performed for control and co-culture group. (**B)** Volcano plot showing significantly upregulated (red), downregulated (green), and non-significant (blue) genes. (**C)** Hierarchical clustering of the gene expression heatmap reveals distinct group-specific patterns. Rows represent genes, columns correspond to samples, and the color scale indicates expression levels (red-high, green-low)

**Fig. 4:**
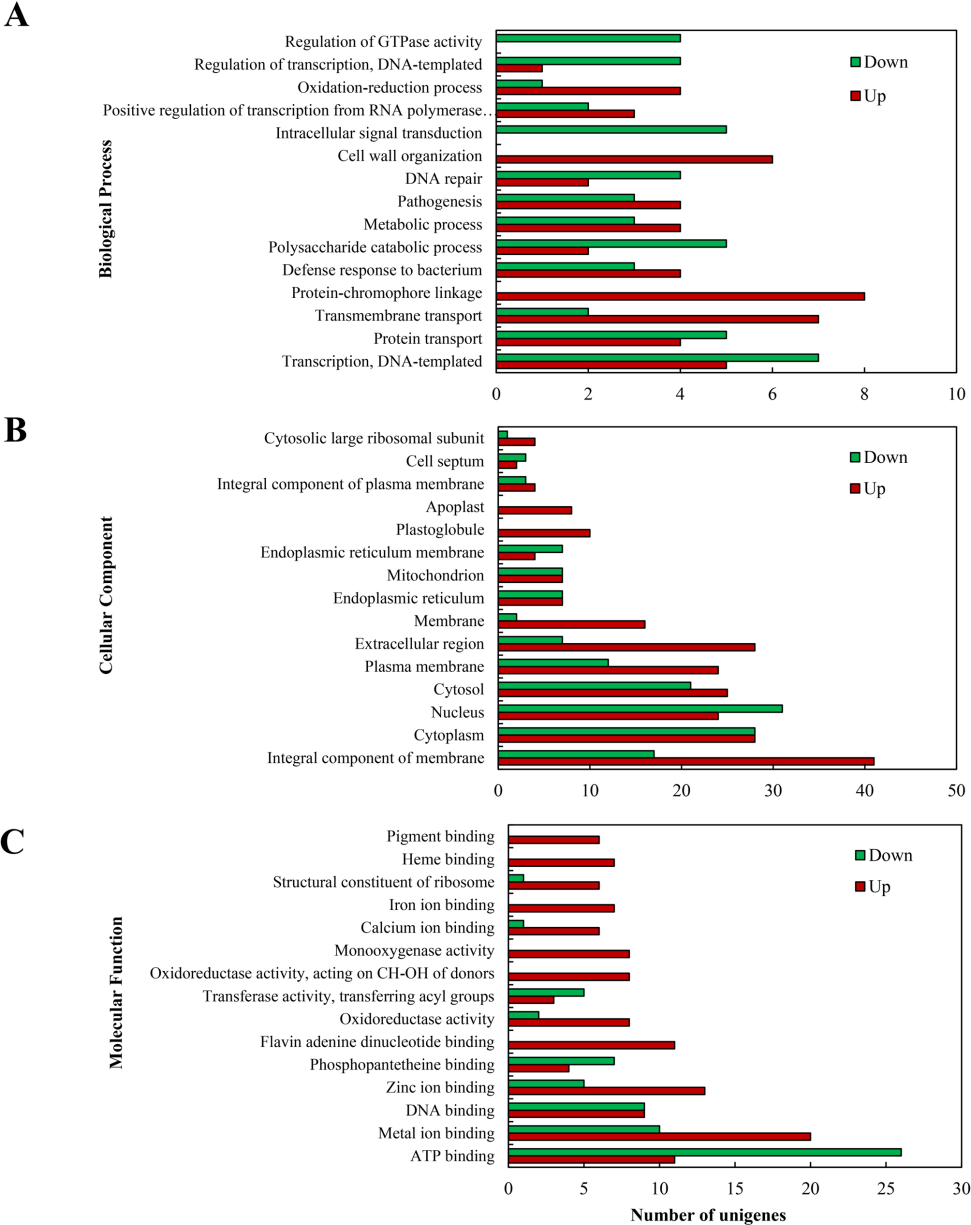
Gene ontology classification of DEGs in *Shiraia* sp. S9 after 24 h co-culture with *Pseudomonas fulva* SB1. (**A)** Biological process. (**B)** Cellular component. (**C)** Molecular function. The cultural condition is the same as specified in Fig. 3

**Fig. 5:**
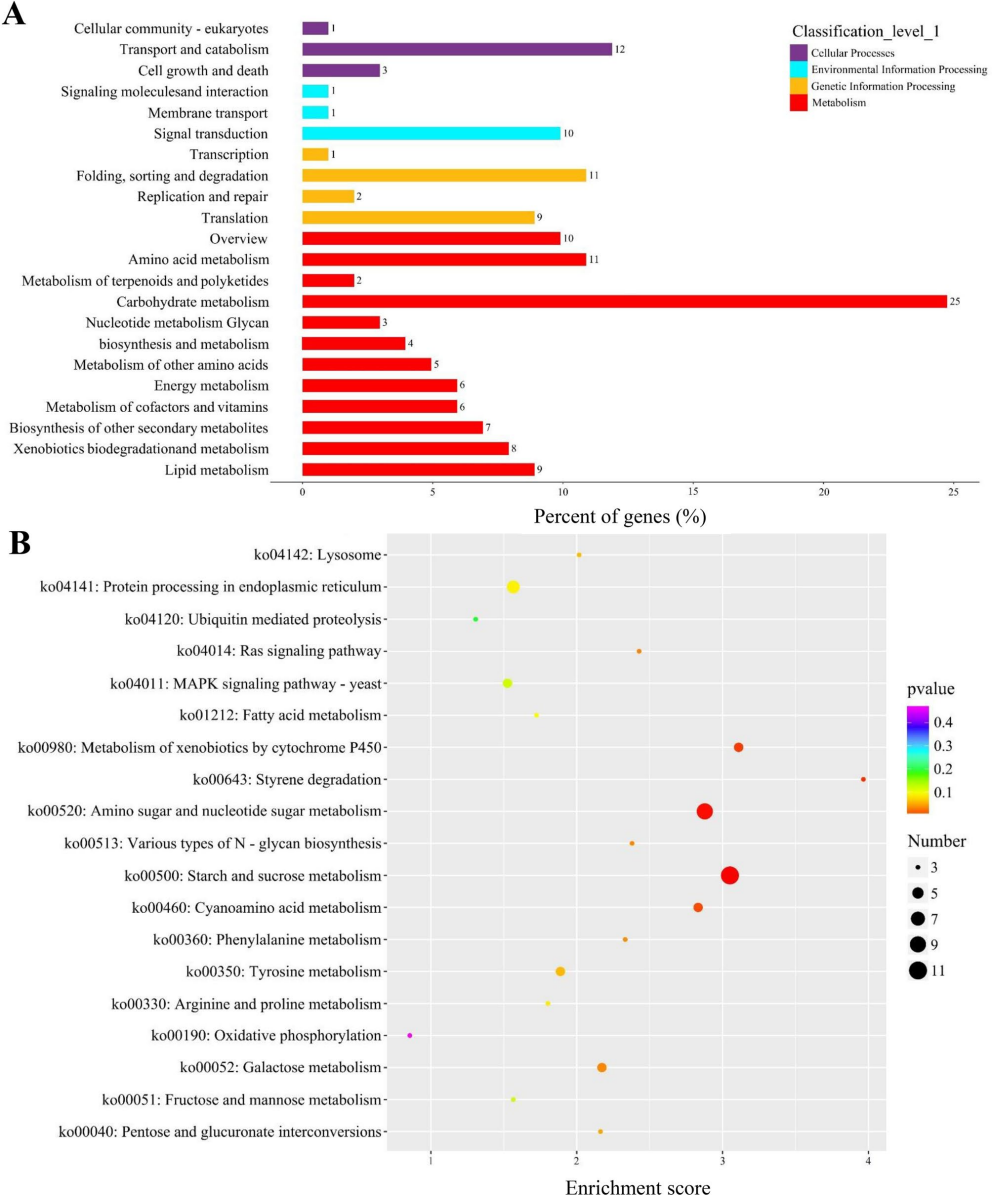
KEGG pathway analysis of DEGs in *Shiraia* sp. S9 during co-culture with *Pseudomonas fulva* SB1 at 24 h. (**A)** Functional distribution of DEGs across primary KEGG categories: metabolism, genetic information processing, environmental information processing, and cellular processes. (**B)** Significant enrichment of DEGs in top KEGG pathways. The cultural condition is the same as specified in Fig. 3

To explore the molecular mechanisms underlying PQ induction by *P. fulva* SB1, transcriptome analysis was conducted on *Shiraia* sp. S9 mycelia after 24 h of co-culture with the live bacterium (contact system). Compared to the fungal monoculture, a total of 646 DEGs were identified, including 445 upregulated and 201 downregulated genes (Fig. 3A). The volcano plot (Fig. 3B) and the hierarchical cluster (Fig. 3C) illustrated the overall distribution of DEGs, with a notable proportion exhibiting significant upregulation and downregulation. Gene Ontology (GO) annotation assigned 737,631, and 672 unigenes to “biological processes”, “cellular components”, and “molecular functions” category, respectively (Fig. 4). Dominant “biological processes” included DNA-templated transcription (12 unigenes), protein transport (9), and transmembrane transport (9), collectively indicating bacterial induction of transcriptional and transport machinery (Fig. 4A). “Cellular component” annotations highlighted integral membrane proteins (58 unigenes), cytoplasmic (56), and nuclear localization (55) (Fig. 4B), while “molecular functions” featured ATP-binding (37), metal ion binding (30), and DNA-binding (18) activities, demonstrating broad activation of catalytic and regulatory functions (Fig. 4C). KEGG pathway analysis showed 65% of DEGs enriched in metabolism, with carbohydrate metabolism (26% of metabolic DEGs) being predominant (Fig. 5A, Table S2), followed by “Genetic Information Processing” (16%), “Cellular Processes” (11%), and “Environmental Information Processing” (8%). Key upregulated pathways included starch/sucrose metabolism (ko00500) and amino sugar/nucleotide sugar metabolism (ko00520), alongside significant alterations in Ras signaling (ko04014), fatty acid metabolism (ko01212), and redox homeostasis pathways (Fig. 5B), collectively indicating profound metabolic rewiring during contact elicitation (Fig. 5B, Table S2).

### Transcriptional regulation of oxidative stress defense and ROS generation

Among the 45 most significantly enriched DEGs (Table S3), key functional categories responsive to *P. fulva* SB1 addition included protein serine/threonine kinase activity (GO:0004674), intracellular signal transduction (GO:0035556), response to low light intensity stimulus (GO:0009645), small GTPase mediated signal transduction (GO:0007264), MAP kinase kinase kinase activity (GO:0004709), response to jasmonic acid (GO:0009753), Ras protein signal transduction (GO:0007265), response to antibiotic (GO:0046677), response to wounding (GO:0009611), response to heat (GO:0009408). Transcriptional activation was observed for critical stress-responsive regulators (Table 1): Ras-related protein (*RAB5A*, comp22429_c0_seq1), 26 S proteasome regulatory complex/ATPase (*RPT4*, comp6671_c0_seq3), serine/threonine-protein kinase (*PPK6*, comp5012_c0_seq2), L-asparaginase 2–4 (*ASP24*, comp16966_c0_seq1).

**Table 1:** Examples of stress- and defense-related DEGs in *Shiraia* sp. S9 in response to co-culture with *Pseudomonas fulva* SB1. The cultural condition is the same as specified in Fig. 3

Unigene ID	Up/down	Fold change	Description [database id]
Response to various stress			
comp22429_c0_seq1	Up	Inf	Ras-related protein Rab-5 A, RAB5A [Q86JP3]
comp6671_c0_seq3	Up	Inf	26 S proteasome regulatory complex/ATPase, RPT4 [KOG0651]
comp5012_c0_seq2	Up	7.52	Serine/threonine-protein kinase, PPK6 [Q9UTH3]
comp16966_c0_seq1	Up	2.12	L-asparaginase 2–4, ASP24 [P0CX79]
comp8104_c0_seq1	Up	1.54	Ankyrin-3, ANK3 [G5E8K5]
Defense response-oxidative stress			
comp6358_c0_seq1	Up	Inf	Oxygen-evolving enhancer protein 1–1, PSBO1 [P23321]
comp20280_c0_seq1	Up	Inf	Peroxisomal membrane protein, PX11B [Q00317]
comp14615_c0_seq1	Up	Inf	Catalase-3, CATA3 [Q42547]
comp14736_c0_seq1	Up	10.22	NADPH oxidase 1, NOX1 [Q9WV87]
comp11049_c0_seq1	Up	5.27	GMC oxidoreductase family, GMC [KOG1238]
comp2357_c0_seq1	Up	1.69	Glutathione peroxidase activity, GPX [GO:0004602]
Defense response to microbe			
comp10609_c0_seq1	Up	Inf	Predicted carbonic anhydrase involved in protection against oxidative damage, PCA [KOG1578]
comp19264_c0_seq1	Up	Inf	Beta carbonic anhydrase 1, BCA1 [P27140]
comp5488_c0_seq1	Up	Inf	Oxygen-evolving enhancer protein 2 − 1, PSBP1 [Q42029]
comp7880_c0_seq1	Up	2.87	Short chain dehydrogenase family protein, SDFP [EXV01934.1]
comp16906_c0_seq1	Up	2.86	Effector protein, PEVD1 [G0Y276]
Phagosome formation			
comp22381_c0_seq1	Up	Inf	V-type proton ATPase subunit c’’2, vATPase [EAZ12652.1]
comp22429_c0_seq1	Up	Inf	GTPase Rab5 [KOG0092]

The introduction of live *P. fulva* SB1 further triggered a pronounced oxidative defense response in *Shiraia* sp. S9, with DEGs predominantly enriched in oxidoreductase activity (GO:0016491), oxygen transporter activity (GO:0005344), cellular response to hyperoxia (GO:0071455), negative regulation of SREBP signaling pathway by positive regulation of transcription factor catabolic process in response to increased oxygen levels (GO:1901487), oxygen binding (GO:0019825), respiratory chain (GO:0070469) (Table S3). Notably, oxygen-evolving enhancer protein 1–1 (*PSBO1*, comp6358_c0_seq1), peroxisomal membrane protein (*PX11B*, comp20280_c0_seq1), and catalase-3 (*CATA3*, comp14615_c0_seq1) were exclusively expressed in the co-culture, while concurrent upregulation occurred for NADPH oxidase (*NOX1*, comp14736_c0_seq1), and antioxidant enzymes glutathione peroxidase (*GPX*, comp2357_c0_seq1) and glucose-methanol-choline oxidoreductase (*GMC*, comp11049_c0_seq1) (Table 1).

**Fig. 6:**
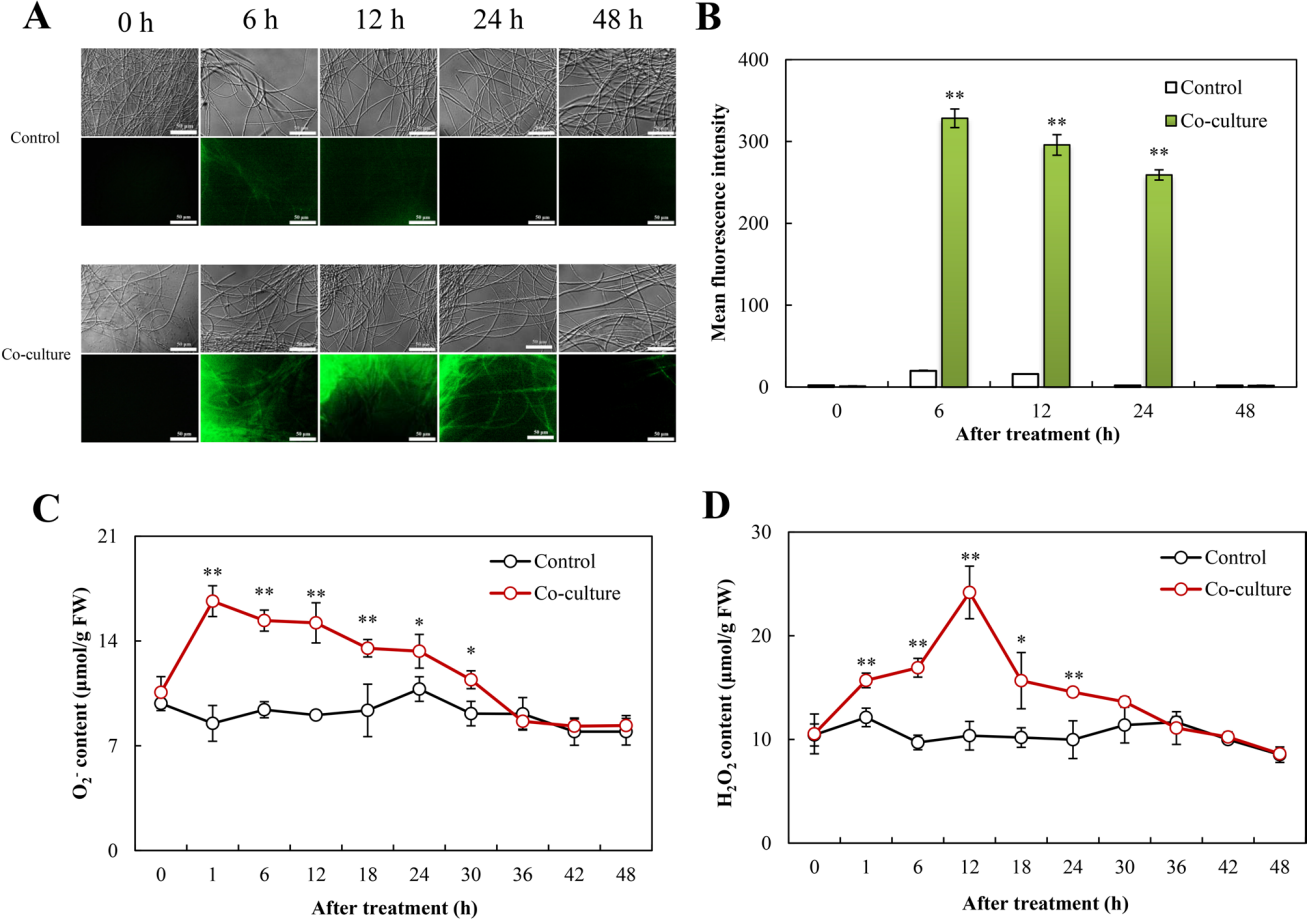
*Pseudomonas fulva* SB1 induced ROS burst and oxidative stress in *Shiraia* sp. S9 during co-culture. (**A**) Representative fluorescence microscopy images of intracellular ROS detected by DCFH-DA staining. Scale bars = 50 μm. (**B**) Quantitative analysis of total ROS levels based on DCFH-DA fluorescence intensity. (**C)** O₂^•⁻^ and (**D**) H_2_O_2_ levels measured in co-cultured S9 hypha. The cultural condition is the same as specified in Fig. 3. *p < 0.05, **p < 0.01 versus control group

**Fig. 7:**
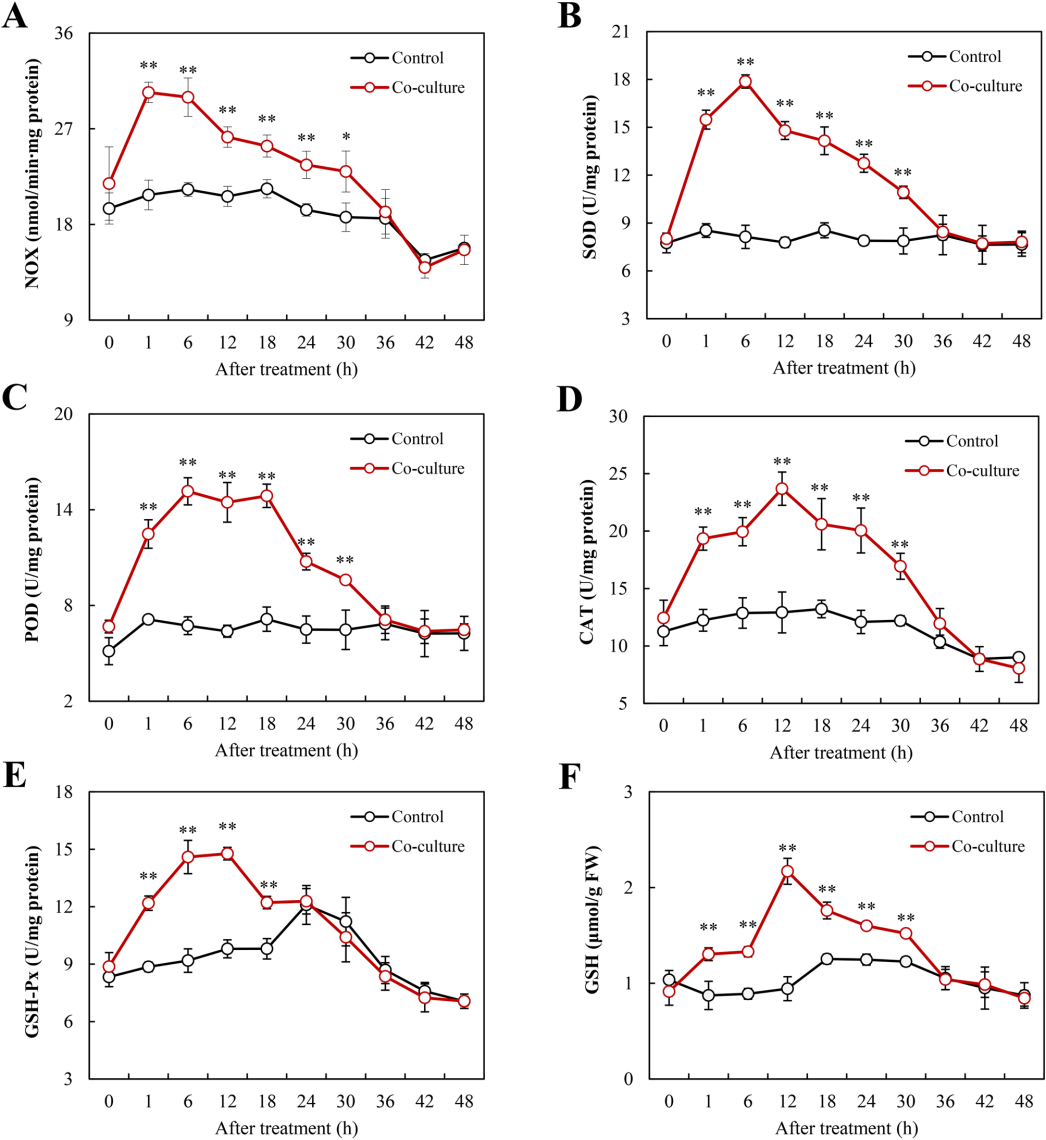
*Pseudomonas fulva* SB1 altered (**A**) NOX, (**B**) SOD, (**C**) POD, (**D**) CAT, (**E**) GSH-Px, and (**F**) GSH levels in *Shiraia* sp. S9 hypha under co-culture stress. The cultural condition is the same as specified in Fig. 3. *p < 0.05, **p < 0.01 versus control group

Bacterium-induced ROS generation was confirmed by intense green fluorescence in DCFH-DA-stained mycelia during early interaction (6–24 h) (Fig. 6A, B), peaking significantly at 6 h before subsiding to baseline by 48 h. This response featured an immediate superoxide anion (O₂^·−^) spike (16.7 µmol/g fresh weight (FW) at 1 h, Fig. 6C) followed by sustained H_2_O_2_ elevation (peaking at 24.2 µmol/g FW by 12 h with about 46–54% increase through 18–24 h, Fig. 6D). Concomitantly, NOX activity peaked at 1 h (30.4 nmol/min·mg protein), a 46% increase above control (Fig. 7A), while antioxidant enzymes showed sequential activation: SOD surged 2.2-fold (17.9 U/mg protein) at 6 h (Fig. 7B), POD maximized at 6 h (15.2 U/mg protein; 2.3-fold; Fig. 7C), CAT peaked at 12 h (23.7 U/mg protein; 1.8-fold; Fig. 7D), and GSH-Px reached 14.8 U/mg protein at 12 h (Fig. 7E), accompanied by a 2.3-fold increase in reduced GSH (2.2 µmol/g FW, Fig. 7F).

### Bacterium-induced cell membrane permeabilization

Cellular component analysis revealed membrane-centric remodeling, with significant enrichment for “integral component of membrane” (GO:0016021), “membrane” (GO:0016020), and “integral component of plasma membrane” (GO:0005887) (Fig. 4B). This restructuring coincided with transcriptional downregulation of saturated fatty acid biosynthesis genes-including *FAS1* (S-acyl fatty acid synthase thioesterase, comp2209_c0_seq2) and *FAS2* (3-oxoacyl-[acyl-carrier protein] synthase, comp19264_c0_seq1) - alongside coordinated upregulation of membrane fluidity modulators and lipid catabolism-related genes (Table S4).

**Fig. 8:**
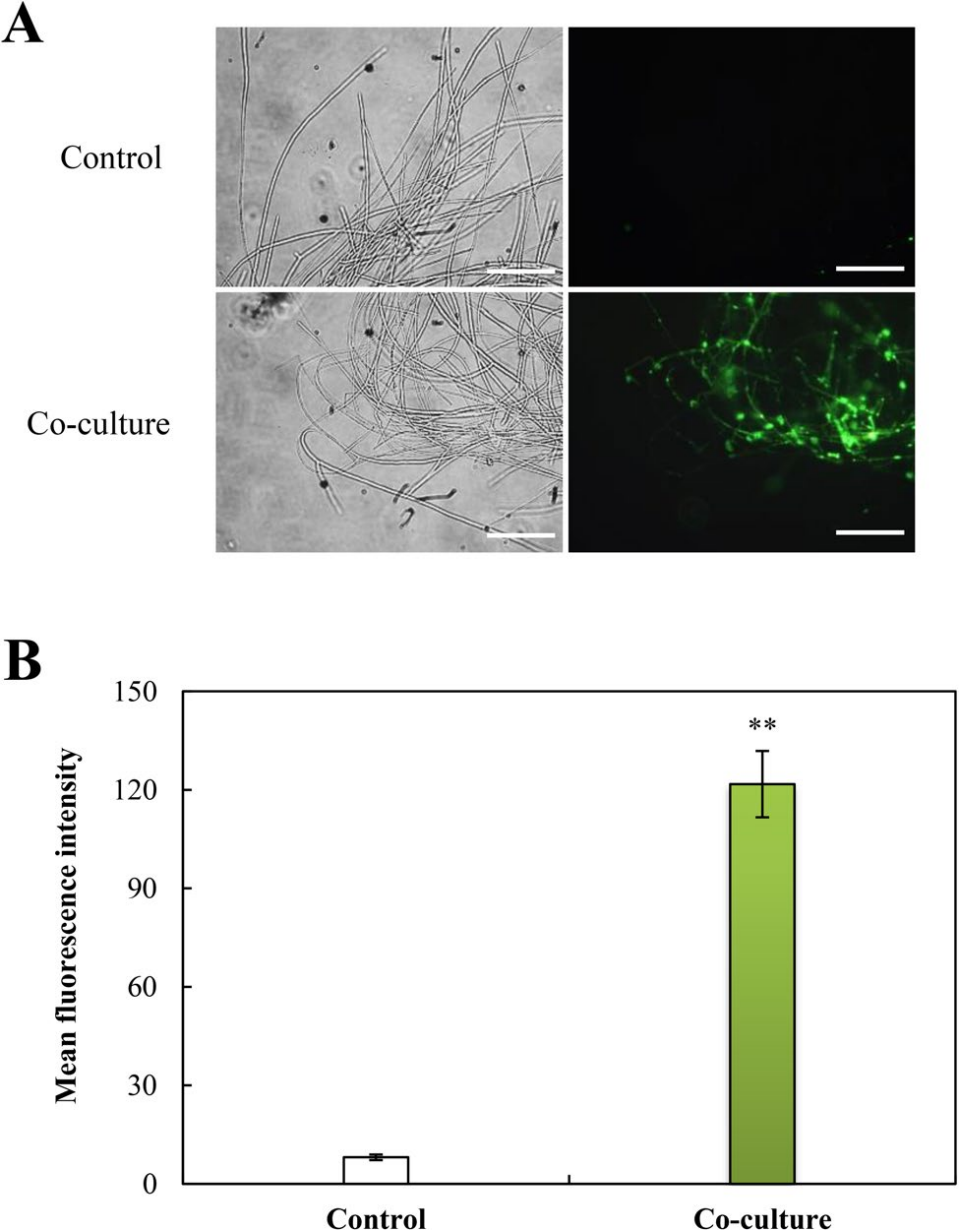
*Pseudomonas fulva* SB1 altered membrane integrity in *Shiraia* sp. S9 during co-culture. (**A**) Representative fluorescence microscopy images of SYTOX Green-stained *Shiraia* sp. S9 hyphae. Scale bars = 50 μm. (**B**) Quantification of SYTOX Green fluorescence intensity indicating membrane permeabilization. The cultural condition is the same as specified in Fig. 3. **p < 0.01 versus control group

Functional validation demonstrated substantial membrane permeabilization in *Shiraia* sp. S9 hyphae during co-culture with *P. fulva* SB1, as evidenced by SYTOX Green fluorescence intensity increasing about 15-fold relative to controls (Fig. 8A, B). Lipidomic profiling further identified significant compositional shifts: saturated fatty acids (SFAs) decreased markedly - pentadecanoic acid (C15:0, −23%), stearic acid (C18:0, −11%), and arachidic acid (C20:0, −28%) compared to controls, while unsaturated fatty acids (UFAs) increased substantially, notably linoleic acid (C18:2, + 48%), α-linolenic acid (C18:3, + 33%), and eicosadienoic acid (C20:2, + 29%) (Table 2). Collectively, these alterations elevated the UFAs/SFAs ratio by 54%, indicating enhanced membrane fluidity during bacterial elicitation.

**Table 2:** *Pseudomonas fulva* SB1 modulated fatty acid composition in *Shiraia* sp. S9 hyphal membrane during co-culture. The cultural condition is the same as specified in Fig. 3

Fatty acid composition	% Total fatty acid	
	Control	Co-culture
C14:0	0.62 ± 0.08	0.61 ± 0.09
C15:0	0.56 ± 0.02	0.43 ± 0.02**
C16:0	29.05 ± 2.20	28.03 ± 1.95
C16:1	0.46 ± 0.03	0.47 ± 0.08
C17:0	1.46 ± 0.14	1.45 ± 0.05
C18:0	18.02 ± 1.09	16.10 ± 0.25*
C18:1	3.36 ± 0.39	4.07 ± 1.06
C18:2	24.23 ± 4.19	35.92 ± 1.56*
C18:3	0.66 ± 0.10	0.88 ± 0.06*
C20:0	0.50 ± 0.08	0.36 ± 0.03*
C20:2	0.34 ± 0.02	0.44 ± 0.02**
C22:0	0.42 ± 0.09	0.39 ± 0.05
C24:0	0.34 ± 0.04	0.29 ± 0.06
Unsaturated/saturated fatty acid ratio^a^	0.57 ± 0.07	0.88 ± 0.03**

^a^The ratio was calculated as (C16:1 + C18:1 + C18:2 + C18:3 + C20:2) / (C14:0 + C15:0 + C16:0 + C17:0 + C18:0 + C20:0 + C22:0 + C24:0)

### Transcriptional regulation of HA biosynthesis

**Fig. 9:**
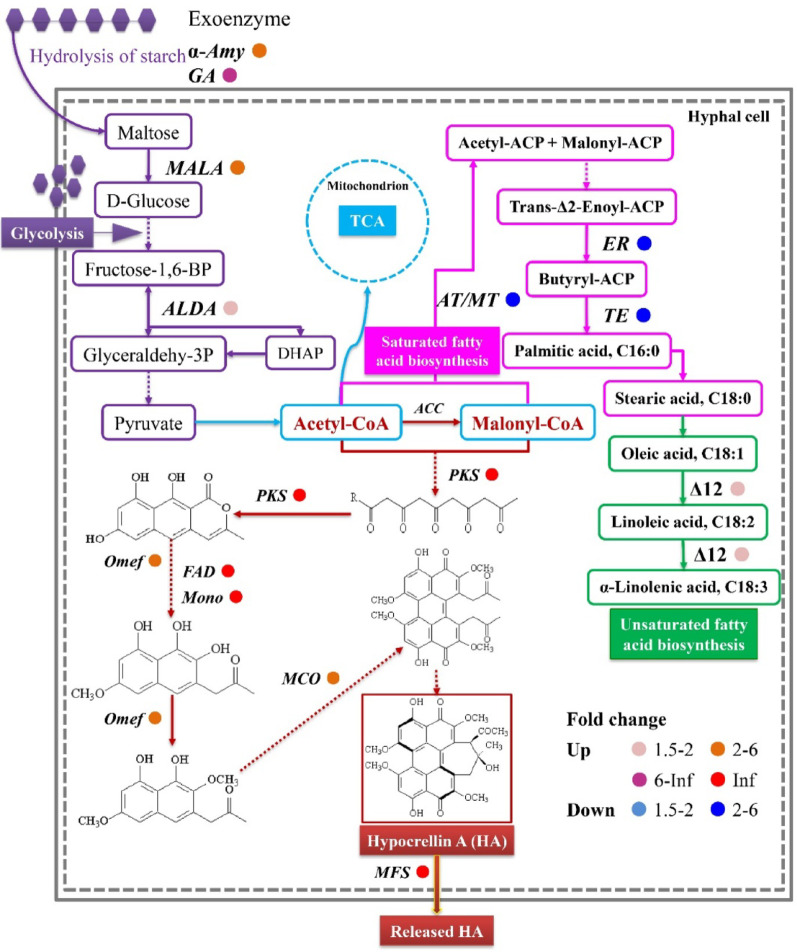
Regulatory network analysis linking hypocrellin biosynthesis with central metabolic pathways in *Shiraia* sp. S9 under *Pseudomonas fulva* SB1 induction. The cultural condition is the same as specified in Fig. 3. DEGs were filtered with |FC| ≥ 1.5 and *p* < 0.05. Simplified pathways omit intermediate steps and compounds. More information about enzymatic annotations are given in Table S5

Given that HA, an effective PDT agent with anticancer and antimicrobe activity (Bao et al. 2023) constituted approximately 58% of total PQs (Fig. 1) and its biosynthetic pathway is well-established (Yang et al. 2014; Lei et al. 2017; Ren et al. 2020), we investigated how *P. fulva* SB1 co-culture modulates HA pathway gene expression. “Carbohydrate metabolism” (Fig. 5A), particularly starch/sucrose degradation coupled with glycolysis (Embden-Meyerhof-Parnas pathway, EMP), supplied essential carbon precursors (Fig. 9). This was evidenced by accelerated glucose consumption in co-culture (Fig. S3) and significant upregulation of gene expressions for starch-hydrolyzing enzymes: α-amylase gene (*α-Amy*) expression peaked at 5.6-fold induction, while glucoamylase (*GA*) and maltase (*MALA)* gene expression increased 6.1-fold and 2.0-fold, respectively (Table S5). Within EMP, aldolase (*ALDA*) transcription rose 2.2-fold, enhancing pyruvate production for conversion to acetyl-CoA and malonyl-CoA as key HA precursors. Concurrently, fatty acid synthase (*FAS*) components (acetyltransferase, *AT*; malonyltransferase, *MT*; enoyl-ACP reductase, *ER*; thioesterase, *TE*) (Table S5) showed over 2.0-fold downregulation (Fig. 9).

Among 27 unigenes annotated to the hypocrellin biosynthetic gene cluster (Table S5), over 88% were upregulated during co-culture. Critically, polyketide synthase (*PKS*, comp3972_c0_seq2), FAD-linked oxidoreductase (*FAD*, comp21370_c0_seq1), and monooxygenase (*Mono*, comp12337_c0_seq1) were exclusively induced (Fig. 9; Table 3). Furthermore, 18 unigenes associated with the extracellular transport of hypocrellins were annotated, predominantly encoding major facilitator superfamily (*MFS*) and ATP-binding cassette (*ABC*) transporters (Table S5). The expressions of two *MFS* transporters (comp20442_c0_seq1 and comp21046_c0_seq1) were enhanced significantly during the co-culture (Fig. 9; Table 3). To validate above-mentioned changes of transcriptomic levels, 19 uigenes were selected for qPCR verification (Table S1). Strong concordance between RNA-seq and qPCR data confirmed transcriptional reliability (Fig. S4).

**Table 3:** Examples of DEGs associated with hypocrellin biosynthesis and transport in *Shiraia* sp. S9 during co-culture with *Pseudomonas fulva* SB1. The cultural condition is the same as specified in Fig. 3

Unigene ID	Up/Down	Fold change	Description [database id]	
Laccase-like multicopper oxidase (*MCO*)				
comp9051_c0_seq1	Up	2.08	Multicopper oxidase [KOG1263]	
comp13841_c0_seq1	Down	1.57	Laccase 2 [Q96WM9]	
Polyketide synthase (*PKS*)				
comp3972_c0_seq2	Up	Inf	Polyketide synthase [AIW00658.1]	
comp5814_c0_seq1	Down	1.88	Polyketide synthase 1 [F1LKH6]	
O-methyltransferase (Omef)				
comp14092_c0_seq1	Up	5.18	*O*-methyltransferase tpcA [Q4WQZ7]	
comp13993_c0_seq1	Up	2.79	Demethylsterigmatocystin 6-*O*-ethyltransferase [Q9UQY0]	
FAD/FMN-dependent oxidoreductase (*FAD*)				
comp21370_c0_seq1	Up	Inf	FAD-linked oxidoreductase ZEB1 [A0A0E0RTV6]	
comp12437_c2_seq1	Up	1.83	Uncharacterized FAD-linked oxidoreductase ARB_02372 [D4B1P2]	
Hydroxylase (*Hyd*)				
comp12205_c0_seq36	Up	2.79	Aromatic hydroxylase fmpF [Q4WD48]	
comp15341_c0_seq1	Up	2.23	Phenylacetate 2-hydroxylase [Q9Y7G5]	
Monooxygenase (*Mono*)				
comp12337_c0_seq1	Up	Inf	Monooxygenase activity [GO:0004497]	
comp14636_c0_seq1	Up	8.99	Cytochrome P450 monooxygenase patI [A1CFL6]	
Major facilitator superfamily (*MFS*)				
comp20442_c0_seq1	Up	Inf	MFS general substrate transporter [OAL04382.1]	
comp21046_c0_seq1	Up	Inf	MFS general substrate transporter [OAL02113.1]	
comp11851_c0_seq1	Up	4.08	MFS general substrate transporter [OAL43313.1]	
comp11507_c0_seq11	Up	2.41	Predicted transporter (major facilitator superfamily) [KOG0254]	
ATP-binding cassette transporter (*ABC*)				
comp16550_c0_seq1	Down	1.94	ABC transporter [AAN28699.3]	
comp16732_c0_seq1	Down	1.78	ZEB2-regulated ABC transporter 1 [I1RL06]	
comp1890_c0_seq1	Down	1.62	Multidrug/pheromone exporter, ABC superfamily [KOG0055]	
Metabolite transport protein				
comp14226_c0_seq1	Up	1.92	Probable metabolite transport protein C1271.09, YHM9 [O94342]	
comp16138_c0_seq1	Up	1.62	Permease of the drug/metabolite transporter superfamily, DMT [KOG4510]	

### Regulatory role of ROS in HA biosynthesis and secretion

**Fig. 10:**
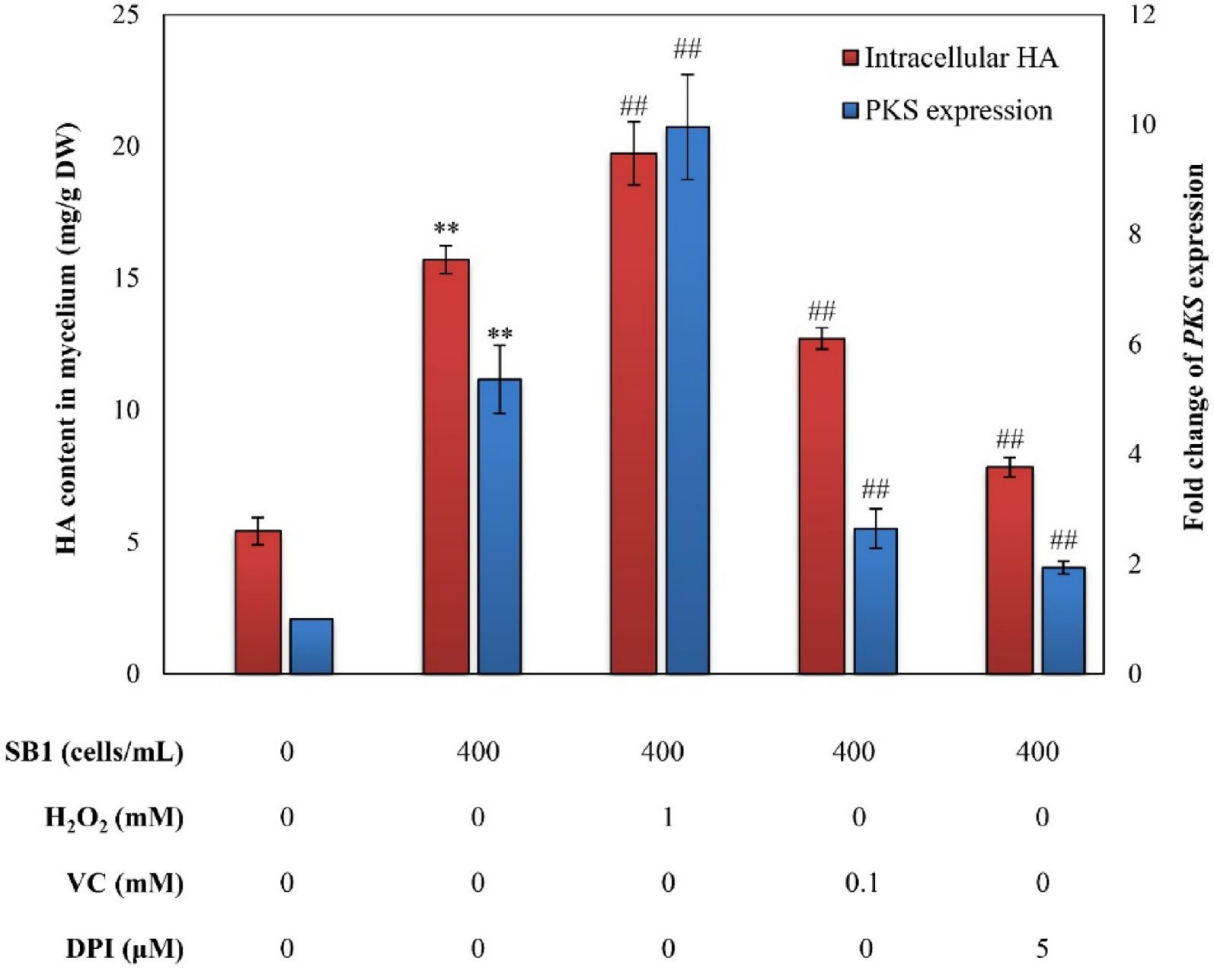
ROS-dependent modulation of HA biosynthesis and *PKS* expression in *Shiraia* sp. S9 during co-culture with *Pseudomonas fulva* SB1. The co-culture condition is the same as specified in Fig. 3. H_2_O_2_, VC and DPI were added 1 h prior to SB1 treatment respectively. **p < 0.01 versus control group without any treatments. ^##^*p* < 0.01 versus co-culture group

To elucidate oxidative stress involvement in *P. fulva* SB1-mediated HA biosynthesis, *Shiraia* sp. S9 cultures were preconditioned with H_2_O_2_ (ROS donor), VC (ROS scavenger), or DPI (NOX inhibitor) prior to bacterial addition. While treatments did not significantly alter fungal biomass (Fig. S5A), they exerted distinct modulatory effects (Fig. S5B). *P. fulva* SB1 or H_2_O_2_ treatment alone elevated HA yields to 2.9-fold or 1.7-fold of axenic controls, respectively, whereas combined H_2_O_2_-*P. fulva* SB1 treatment synergistically enhanced HA production to 19.7 mg/g DW, representing 26% and 117% increase over individual *P. fulva* SB1 or H_2_O_2_ treatment (Fig. 10). Conversely, VC or DPI substantially attenuated bacterium-induced HA production. Parallel upregulation of *PKS* expression under ROS-elevating conditions confirmed ROS-mediated transcriptional regulation of HA biosynthesis (Fig. 10).

**Fig. 11:**
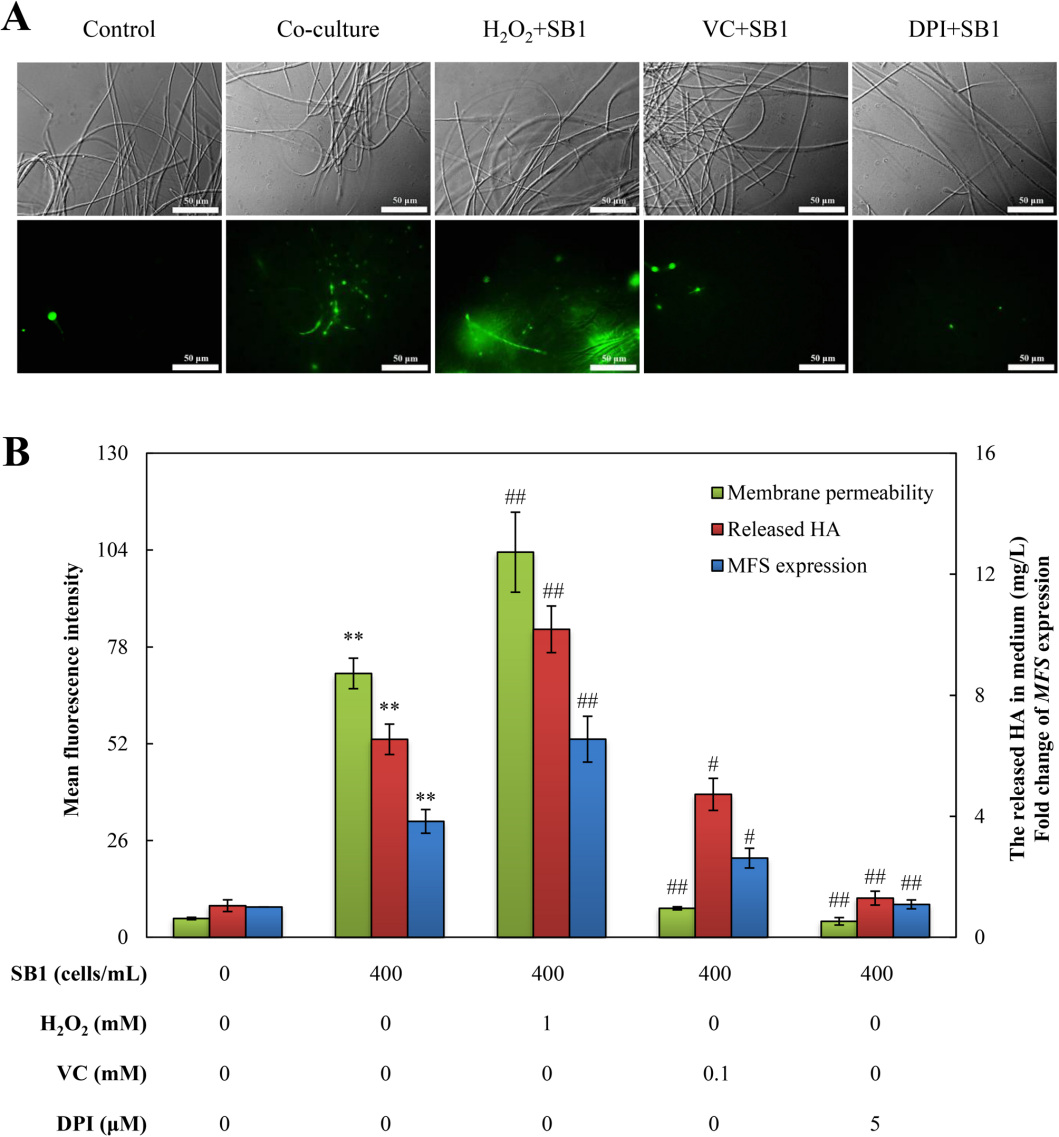
ROS signaling mediated membrane integrity and HA transport in *Shiraia* sp. S9 co-cultured with *Pseudomonas fulva* SB1. (**A**) Representative fluorescence microscopy images of SYTOX Green-stained S9 hypha showing ROS-dependent membrane permeabilization. Scale bars = 50 μm. (**B**) Quantitative analysis of fluorescence intensity, extracellular HA, and *MFS* expression levels under ROS modulation. The co-culture condition is the same as specified in Fig. 3. H_2_O_2_, VC and DPI were added 1 h prior to SB1 treatment respectively. **p < 0.01 versus control group without any treatments. ^#^*p* < 0.05, ^##^*p* < 0.01 versus co-culture group

Assessment of ROS involvement in extracellular HA transport revealed that *P. fulva* SB1 increased membrane permeability (quantified by SYTOX Green fluorescence), an effect potentiated by preconditioning but suppressed by VC and DPI to 11% and 6% of bacterial-treated levels, respectively (Fig. 11A, B). Concomitantly, extracellular HA secretion mirrored these trends: H_2_O_2_-*P. fulva* SB1 co-treatment elevated secreted HA to 10.2 mg/L (55% higher above *P. fulva* SB1 alone), while VC and DPI reduced it by 28% to 4.7 mg/L, and by 80% to 1.3 mg/L, respectively (Fig. 11B). The *MFS* expression showed congruent modulation under these treatments, confirming ROS-dependent regulation of HA extrusion.

## Discussion

*Shiraia* fruiting bodies represent the main resources for “Zhu Huang” in traditional Chinese medicine and the clinically significant hypocrellin-based photodynamic therapy agents (Fiegler-Rudol et al. 2025). PQ yield depends critically on both PQ biosynthetic capacity in *Shiraia* fruiting bodies (ascostromata) (Zhang et al. 2024a, b). The reddish *Shiraia* ascostromata (1–4 cm long × 0.5–2.5 cm wide) composed of wide and woven hyphae of textura intricate, where globose or subglobose perithecia were immersed in their peripheral layer (Liu et al. 2013). Our prior cultivation and Illumina sequencing revealed *Pseudomonas* as a predominant endophytic genus within *Shiraia* fruiting bodies (Ma et al. 2019a), with bacterial colonization observed on asci and ascospore surfaces (Fig. 2A). More recently, we found that endophytic *P. fulva* SB1 could secret matrix exopolysaccharides (EPS) (Zhou et al. 2023), which facilitate the formation of biofilms on the fungal surfaces (Shin et al. 2025). In this study, the intimate physical contact between the bacterium and hyphae of host *Shiraia* sp. S9 was observed (Fig. 2B). Cell-to-cell contact between bacteria and fungi are well-documented in agricultural symbioses (e.g., arbuscular mycorrhizal-bacterial and *Rhizopus*-*Mycetohabitans* symbioses) and animals (including humans) health (e.g., *Candida*-*Staphylococcus*, *Streptococcus* or *Pseudomonas*) (Steffan et al. 2020). The attachment of *S. aureus* to *C. albicans* hyphae accelerated the invasion and disseminate throughout host tissue, resulting in greater morbidity and mortality induced by the infection (Schlecht et al. 2015). As microbes co-exist in plant rhizosphere, the intimate fungal-bacterial associations influence plant health (Partida-Martinez et al. 2005, Frey-Klett et al. 2011). However, the functional significance of bacterial and fungal contact in fungal fruiting bodies, particularly regarding secondary metabolism, remains largely unexplored. Our findings established that physical contact was not merely a prerequisite but the primary ecological trigger for a heightened fungal defense response. Unlike volatile signals (Zheng et al. 2025), which facilitated long-distance communication, direct contact necessitated an immediate and potent countermeasure. The specific imperative for contact in extracellular PQ secretion (Fig. 2C, D) suggested the fungus employed these photoactive compounds as a defensive barrier at the hyphal surface, potentially to mitigate bacterial colonization or biofilm formation. A critical question is whether bacterial metabolites, rather than contact itself, mediate the elicitation. Our dialysis bag experiments conclusively showed that diffusible factors are insufficient. The exchange of small molecules between the fungus and bacterium in the non-contact system resulted only in a modest increase in intracellular PQ content (Fig. 2), failing to trigger the hallmark response of enhanced secretion. This indicates that while metabolic exchange may occur, the physical interaction itself is the non-redundant signal that activates the comprehensive defense program, including the profound membrane and transport changes necessary for secretion. While our findings demonstrate that *P. fulva* SB1 can act as a potent elicitor of PQ metabolism under laboratory co-culture conditions, several limitations must be considered when extrapolating this role to a natural setting. First, the elicitation effect may be strain-specific; not all endophytic *Pseudomonas* isolates tested exhibited the same strong stimulatory effect (Fig. 1B), highlighting the functional diversity within the fruiting body microbiome. Second, our study was conducted in a simplified axenic laboratory system, which, while powerful for identifying mechanistic pathways, lacks the complexity of the natural fungal fruiting body environment where multiple microbial taxa and host factors interact simultaneously. The net effect on the host fungus *in situ* likely results from a balance of synergistic and antagonistic interactions within the entire microbial consortium. Therefore, our results position *P. fulva* SB1 as a compelling model for demonstrating the potential of endophytes to act as cryptic metabolic facilitators. Recently both ^13^C quantitative stable isotope probing (qSIP) and cross-domain network analysis were employed to reveal intricate carbon exchange and direct interactions between bacteria and fungi in the hyphosphere (Slanzon et al. 2025). Future studies isolating a wider array of bacterial strains, employing synthetic microbial communities within more complex model systems and incorporating qSIP analysis will be essential to validate the ecological relevance and generalizability of this fascinating interaction.

Our findings on contact-dependent elicitation align with recent advances in understanding bacterial-fungal interactions. Genome-wide analyses have revealed that fungi can profoundly alter bacterial fitness through both conserved mechanisms (e.g., modulating micronutrient availability) and species-specific effects (Braat et al. 2022), consistent with the strain-specific effects we observed among *Pseudomonas* isolates (Fig. 1B). Furthermore, the widespread transcriptional rewiring we documented in *Shiraia* mirrors findings from fungal-fungal cocultivation systems, where microbial interactions activate global regulators like the velvet complex, leading to the profound restructuring of secondary metabolism and the production of novel compounds (Wang et al. 2022). The enduring nature of our observed bacterial effects on fungal metabolism may also find parallels in human gut microbiome studies (Seelbinder et al. 2020), where antibiotic-induced disruptions lead to long-lasting alterations in fungal community dynamics and interkingdom relationships. The contact-dependent mechanism complements previously reported volatile-mediated (long distance) interactions in *Shiraia* fruiting bodies (Xu et al. 2022, 2023; Zheng et al. 2025). Given the prevalence of *Pseudomonas* species in fungal fruiting bodies of *Suillus grevillea* (Varese et al. 1996), *A. bisporus* (Braat et al. 2022) and *T*.* matsutake* (Li et al. 2016), their roles in regulating development, medicinal value or food quality of the fruiting bodies (mushrooms) warrants significant consideration. Beyond their biotechnological potential, our findings invite speculation on the ecological significance of contact-mediated PQ induction within the native context of the fruiting body. The compartmentalized structure of the ascostromata, with perithecia embedded in a stroma of fungal tissue (pseudoparenchyma), creates a unique microenvironment where bacterial endophytes like *P. fulva* reside in intimate association with hyphae and developing ascospores (Fig. 2A). In this context, the contact-dependent mechanism we describe may represent a nuanced form of interaction with dual implications. On one hand, the bacterium-induced production and secretion of broad-spectrum antimicrobial PQs could be interpreted as a fungal defense response to a perceived threat of bacterial overgrowth, helping to maintain homeostasis within the reproductive structure and potentially protecting the valuable spore-producing tissues. On the other hand, the fact that the bacterium persists suggests a more complex relationship. The interaction may exist on a mutualistic-parasitic continuum, where a low level of bacterial colonization “primes” or “elicits” the fungal chemical defense system. This could be beneficial for the fungus by enhancing its overall chemical arsenal against other pathogens or predators, while the bacterium may tolerate or even resist the PQs to gain access to a nutrient-rich niche.

While certain bacterial isolates from *T. matsutake* fruiting bodies demonstrated growth-promoting effects, the majority exerted inhibitory effects on host mycelial development (Oh et al. 2018). These antagonistic bacteria exhibited chitinase, cellulase, protease, and lipase activities for degradation of fungal cell wall components, suggesting nutrient competition between symbiotic microorganisms. Moreover, endophytic *Pseudomonas* species isolated the fruiting bodies such as *A. bisporus* and *T. borchii* displayed broad-spectrum antifungal properties (Sbrana et al. 2002; Xiang et al. 2017). Although live *P. fulva* SB1 did not significantly inhibit *Shiraia* biomass (Fig. S5A), it induced some stress responses including the fragmentation of hyphae, the increased conidiation and reduced pellet size (Ma et al. 2019b). The transcriptomic profile painted a picture of a fungus mounting a coordinated defense primed for surface engagement. The significant enrichment of genes related to integral membrane components (GO:0016021) and Ras GTPase signaling (GO:0007265) indicated the perception of contact stress at the plasma membrane and the activation of downstream defense orchestrators (Table 1, Table S3). This is not a general stress response but a targeted reprogramming for biotic confrontation, shifting resources from primary metabolism (e.g., downregulated FAS) to the production and deployment of antimicrobial defense compounds (PQs). Given that ROS production constitutes a fundamental fungal defense mechanism against bacterial antagonists, our transcriptomic data revealed DEGs predominantly to oxidoreductase activity, oxygen transporter activity and cellular responses to increased oxygen levels and oxygen binding (Table S3), with concurrent upregulation of antioxidant enzymes (Table 1). These findings collectively indicate that *P. fulva* SB1 colonization triggers both ROS generation and quenching-a hallmark of fungal immune responses (Aguirre et al. 2006). We proposed a novel model wherein controlled, contact-induced ROS generation acts as a double-edged sword in fungal defense strategy. The initial ROS burst served as a critical secondary messenger to upregulate biosynthetic and transport machinery. Concomitantly, it drove a targeted increase in membrane fluidity (evidenced by the elevated UFA/SFA ratio) that facilitated the efflux of toxic PQs (Table 2; Fig. 8). This regulated permeabilization represented a calculated trade-off, sacrificing minimal membrane integrity to enable the efficient secretion of defensive metabolites against the physically attached bacterium. Since photodynamically active PQs secreted by *Shiraia* exhibited antibacterial properties that may alleviate bacterial stress (Su et al. 2011; Ma et al. 2019b), these findings establish a direct relationship between endophyte colonization and fungal PQ biosynthesis. This study also reveals a previously unrecognized mechanism of endophyte-fungal interaction within fruiting bodies: contact-dependent elicitation mediated by ROS signaling. Unlike volatile-mediated cross-kingdom communication (Zheng et al. 2025), physical contact with *P. fulva SB1* triggered a rapid generation of ROS (the highest O₂^•⁻^ production at 1 h and H_2_O_2_ prodoction at 12 h) (Fig. 6), while it took about 5 d for *Shiraia* cultures treated by volatiles of *Rhodococcus* sp. No. 3 to achieve the highest H_2_O_2_ production (Zheng et al. 2025). This discrepancy was likely due to different responses to bacterial volatiles and physical contact.

Intracellular ROS regulate key developmental processes in fungi, including hülle cell formation and cleistothecial initiation in *Aspergillus nidulans* (Lara-Ortíz et al. 2003), the light-induced perithecial polarity of *Neurospora crassa* (Belozerskaya et al. 2012), and the perithecial/ascus development in *Ophiocordyceps sinensis* fruiting bodies (Tong et al. 2021). In this study, *P. fulva* SB1-enhaced HA production was abolished by ROS scavenger VC and NOX inhibitor DPI, while potentiated by H_2_O_2_ supplementation (Fig. 10), demonstrating ROS mediation of bacterial-induced PQ biosynthesis in the fruiting bodies. The transcriptomic and genomic analysis confirmed EMP and TCA cycle pathways provide essential carbon precursors for PQ biosynthesis in *Shiraia* (Lei et al. 2017; Yang et al. 2014; Ren et al. 2020). Accelerated glucose consumption during co-culture (Fig. S3A) indicated bacterial modulation of central carbon metabolism, supported by upregulation of starch-degrading enzymes: α-amylase (α-*Amy*), glucoamylase (*GA*) and maltase (*MALA*) were upregulated respectively to facilitate starch degradation and glycolysis (Fig. 9). Concurrently, *ALDA*, an EMP rate-limiting enzyme aldolase gene exhibited significant induction, while the gene expressions related to fatty acid synthesis (Table S5), including acetyltransferase (*AT*), malonyltransferase (*MT*), enoyl-ACP reductase (*ER*), and thioesterase (*TE*), exhibited significant downregulation (Fig. 9), redirecting metabolic flux toward acetyl-CoA (key PQ precursor). PQ biosynthesis initiates through PKS-mediated decarboxylative Claisen condensation of acetyl-CoA and malonyl-CoA, with *O*-methyltransferase (Omef) generating PQ backbones (Yang et al. 2014; Ren et al. 2020). Late-stage modifications involve FAD, Mono and MCO responsible for ketone reduction, dimerization and side chain cyclization. During co-culture, all PQ biosynthetic and transporter genes were significantly upregulated (Table 3; Fig. 9), including *MFS* transporters that mediate PQ efflux in *S. bambusicola* (Deng et al. 2017). Critically, *P. fulva* SB1-induced expression of *PKS* (for biosynthesis) and *MFS* (for transport) was suppressed by VC/DPI pretreatment (Figs. 10 and 11), confirming ROS-dependent regulation of the PQ biosynthetic cascade during bacterial-fungal interaction. The broad-spectrum enhancement of multiple PQs (HA, HC, EA, EB, EC) (Fig. 1)—in contrast to the specific HA induction observed in volatile-mediated interactions (Zheng et al. 2025)—suggests that physical contact triggers a maximal defense response. The fungus does not selectively upregulate a single compound but deploys a synergistic cocktail of phototoxic PQs, potentially to increase the efficacy and scope of its antimicrobial defense against a direct physical challenge. This indicates that the regulatory impact of bacterial elicitors is not limited to a single metabolic branch but can orchestrate the coordinated activation of an entire biosynthetic pathway, revealing a more complex layer of microbial interaction within the fruiting body. Collectively, our data support a paradigm where endophytic contact is perceived as an antagonistic challenge, triggering a defined signaling cascade: Contact → NOX-mediated ROS burst → (1) Transcriptional activation of defense genes and (2) Membrane remodeling → Enhanced biosynthesis and targeted secretion of PQs. This mechanism reveals a sophisticated fungal strategy to manage intimate microbial associations within its fruiting body, transforming a potential threat into a trigger for enhanced chemical defense production.

## Conclusions

In summary, this investigation established that dominant *Pseudomonas* isolates from *Shiraia* fruiting bodies significantly enhanced PQ production through direct physical contact with the host fungus *Shiraia* sp. S9. Our findings revealed that such contact reprogramed global gene expression, with pronounced upregulation of defense response pathways and PQ biosynthetic machinery, while triggering early ROS generation (1–6 h post-contact) that mediated fungal membrane permeabilization and coordinated transcriptional activation of both PQ biosynthesis-notably the photodynamically active HA-and secretion mechanisms. These results define a novel elicitation mechanism in which ROS acts as a central signaling node, wherein endophytic colonization stimulates defensive metabolite production within fruiting bodies, providing fundamental insights into bacterial-fungal interactions.

Looking forward, this work opens several compelling avenues for future research. First, the strain-specificity of this phenomenon should be systematically explored by testing whether other *Pseudomonas* species, or even genera, isolated from *Shiraia* and other medicinal fungi can elicit similar PQ induction, which would help determine if this is a conserved mechanism or a unique adaptation of the *P. fulva* SB1 strain. Second, the molecular signaling pathways immediately downstream of the perceived contact signal remain to be fully elucidated. Identifying the fungal membrane receptors or sensors that detect bacterial contact, and the subsequent mitogen-activated protein kinase (MAPK) cascade or calcium signaling that leads to NOX activation and the ROS burst, represents a critical next step. Third, the transcriptional regulators (e.g., specific transcription factors activated by the ROS signal) that directly bind to the promoters of the PQ biosynthetic gene cluster and transporter genes need to be characterized to complete the signaling circuit from membrane to nucleus. Finally, from a biotechnological perspective, our co-culture strategy should be optimized and scaled, for instance, by using immobilized bacterial cells in bioreactors to continuously elicit fungal production, potentially offering a robust and sustainable platform for the industrial-scale production of these valuable photosensitizers, overcoming the inherent yield limitations in axenic fungal cultivation. Thus, beyond revealing a new paradigm for contact-mediated dialogue within the fruiting body microbiome, our work provides a foundational mechanistic understanding for harnessing these ancient fungal-bacterial interactions in innovative co-culture technologies to sustainably produce valuable natural PQ photosensitizers.

## Supplementary Information

Below is the link to the electronic supplementary material.

Supplementary Material 1

Supplementary Material 2

## Data Availability

The datasets generated and analyzed during this study are included in the published article [and its supplementary information files].
