# Supplementary material for: Fruiting body-associated Pseudomonas contact triggers ROS-mediated perylenequinone biosynthesis in Shiraia mycelium culture
Source: Bioresour Bioprocess. 2025 Sep 26;12(1):106. doi: 10.1186/s40643-025-00946-w (PMC12474784; doi:10.1186/s40643-025-00946-w)
Supplement: Supplementary file 2 — Supplementary Material 2 [file 40643_2025_946_MOESM2_ESM.pdf]

Supplementary materials

Fruiting body-associated *Pseudomonas* contact triggers ROS-mediated perylenequinone production in Shiraia mycelium culture

Yan Jun Ma, Xin Ping Li, Jia Hui Li, Li Ping Zheng, Jian Wen Wang

✉ Jian Wen Wang

jwwang@suda.edu.cn

Table S1 Primers and relevant information of reference and target genes. F: forward primer, R: reverse primer.

Table S2 KEGG pathway enrichment of *Shiraia* sp. S9 with *Pseudomonas fulva* SB1 treatment (See Additional file 2).

Table S3 DEGs involved in the response to various stress and defense response of *Shiraia* sp. S9 by *Pseudomonas fulva* SB1 (See Additional file 2).

Table S4 DEGs involved in hypocrellin transmembrane transport of *Shiraia* sp. S9 induced by *Pseudomonas fulva* SB1 (See Additional file 2).

Table S5 DEGs involved in hypocrellin biosynthesis in *Shiraia* sp. S9 under *Pseudomonas fulva* SB1 induction (See Additional file 2).

Fig. S1 Effect of inoculum concentration of *Pseudomonas fulva* SB1 on total PQ production in submerged culture of *Shiraia* sp. S9. *P. fulva* SB1 was inoculated on day 6 of mycelium culture at 150 rpm, 28°C. Total PQ production refers to the sum of the intracellular and extracellular PQs. Different letters above the bars mean significant differences ($p < 0.05$).

Fig. S2 Effects of *Pseudomonas fulva* SB1 on (**A**) mycelium dry biomass and (**B**) total PQ production in submerged culture of *Shiraia* sp. S9 on day 8. *P. fulva* SB1 was inoculated at 400 cells/mL on day 6 of mycelium culture at 150 rpm, 28℃. Statistical comparison between the two groups showed no significance, which is denoted as "ns". $^{**}p < 0.01$ versus "-SB1" group. "-SB1" group refers control group without the bacteria. $^{\#}p < 0.05$ denotes the significant difference between the "+SB" in fungal co-culture and non-contact co-culture with bacteria.

Fig. S3 Time profiles of (**A**) residual sugar and (**B**) medium pH in submerged cultures of *Shiraia* sp. S9 with the addition of *Pseudomonas fulva* SB1. The procedure of co-culture was the same as specified in Fig. 3. The *arrow* represents addition time of *P. fulva* SB1. $^{*}p < 0.05$, $^{**}p < 0.01$ versus control group.

Fig. S4 Validation of the expression levels of genes related to (**A**) growth and development, (**B**) biosynthesis and transport of hypocrellins and (c) oxidoreductase activities of co-culture between *Pseudomona fulva* SB1 and *Shiraia* sp. S9 by qPCR. The cultural condition is the same as specified in Fig. 3.

Fig. S5 ROS-dependent modulation of biomass (**A**) and HA biosynthesis (**B**) in *Shiraia* sp. S9 during co-culture with *Pseudomonas fulva* SB1. The optimized co-culture protocol is the same as shown in Fig. 3. H_2O_2, VC and DPI were added 1 h prior to SB1 treatment respectively. $^{**}p < 0.01$ versus control group without any treatments.

Table S1 The primers of the target genes used for qRT-PCR.

Unigene name	Unigene ID	Gene description	Sequence
Reference	comp12301_c0_seq8	18S ribosomal RNA	F: 5’-ACGCAGCGAAATGCGATAAG-3’<br>R: 5’-CAAATTGTGCTGCGCTCCAA-3’
CRZA	comp4570_c0_seq1	C2H2 finger domain transcription factor [Q4WJ81]	F: 5’-TCACTGTTCTGGCCTTGTCC-3’<br>R: 5’-AACCACACAATCGCATTCGC-3’
AMYA1	comp16299_c0_seq1	Alpha-amylase A type-1/2 [P0C1B3]	F: 5’-AAGAAACTCACTACCGCCCG-3’<br>R: 5’-CCACTACCTGCATTGGTCGT-3’
UBC2	comp9275_c0_seq2	Ubiquitin-conjugating enzyme E2 [P52493]	F: 5’-GCCAAACACCAAACTGCGAT-3’<br>R: 5’-CGCCTTTTGAGGATGGGACT-3’
PLD1	comp15153_c0_seq1	Ascospore-type prospore assembly [GO:0031321]	F: 5’-CACCCTTCGTGTTCGTCTCA-3’<br>R: 5’-GCGAACGCAGCATACTCTTG-3’
CHS6	comp12655_c0_seq1	Chitin synthase 6 [O13395]	F: 5’-TGGGTTCGAGAAGGAACGTG-3’<br>R: 5’-AGATTCCGTGACAACTGCGT-3’
CSN6	comp14169_c0_seq1	Cleistothecium development [GO:0070791]	F: 5’-GATGGCGAGGTTGTCCTTGA-3’<br>R: 5’-AGGTTCGTAAAGGGTGAGCG-3’
PKS	comp3972_c0_seq2	Polyketide synthase [AIW00658.1]	F: 5’-TTCAGCCGAGTGGTTGGTTC-3’<br>R: 5’-TGCTGTCCTGAAACGACTGG-3’
Omef	comp14092_c0_seq1	*O*-methyltransferase tpcA [Q4WQZ7]	F: 5’-AGTAGTGTTGCCTGACTCGC-3’<br>R: 5’-AGTATCACGGAACAGCCAGC-3’
Mono	comp12337_c0_seq1	Monooxygenase activity [GO:0004497]	F: 5’-TTCGAGTTGGAGCACGGATT-3’<br>R: 5’-CACTGGACTTGACGGGTGTT-3’
MCO	comp9051_c0_seq1	Multicopper oxidase [KOG1263]	F: 5’-AGTCGCCCTCCGATACTCTT-3’<br>R: 5’-AAGCATCACATCGTGCCAGA-3’
MFS	comp20442_c0_seq1	MFS general substrate transporter [OAL04382.1]	F: 5’-GCCTTTGAGTCTTCCTCGGT-3’<br>R: 5’-TGCGAACGTGGCTTTCTCTA-3’
YHM9	comp14226_c0_seq1	Probable metabolite transport protein C1271.09 [O94342]	F: 5’-CGCATACTCCTTCGGCATCT-3’<br>R: 5’-ATGCCGTAGTATTGGCCTCG-3’
PPK6	comp5012_c0_seq2	Serine/threonine-protein kinase [Q9UTH3]	F: 5’-GTTCTTTCCCTCGGTACGGG-3’<br>R: 5’-GGGACCATCGGTAAGTGCAA-3’
NOX1	comp14736_c0_seq1	NADPH oxidase 1 [Q9WV87]	F: 5’-GCTTAACTCCGGCCTTGTCT-3’<br>R: 5’-GATTGTTCGGTCGCTGATGC-3’
CATA3	comp14615_c0_seq1	Catalase-3 [Q42547]	F: 5’-CGATTGGCTGGCCGTTATTG-3’<br>R: 5’-GGGATGCCCGTACTATCAGC-3’
GPX	comp2357_c0_seq1	Glutathione peroxidase activity [GO:0004602]	F: 5’-CATCCAGACCCTGCGACTTT-3’<br>R: 5’-GGTGCGCGTTGGTAGTTTTT-3’
GMC	comp11049_c0_seq1	GMC oxidoreductase family [KOG1238]	F: 5’-AACTGGAAATCAGAGGCCCG-3’<br>R: 5’-ACTTCGCTTGTACTCGGCAA-3’
BCA1	comp19264_c0_seq1	Beta carbonic anhydrase 1 [P27140]	F: 5’-CGCTTATCCGTAACGAGCCA-3’<br>R: 5’-GTCGTATGCTTCGGTTCCCA-3’

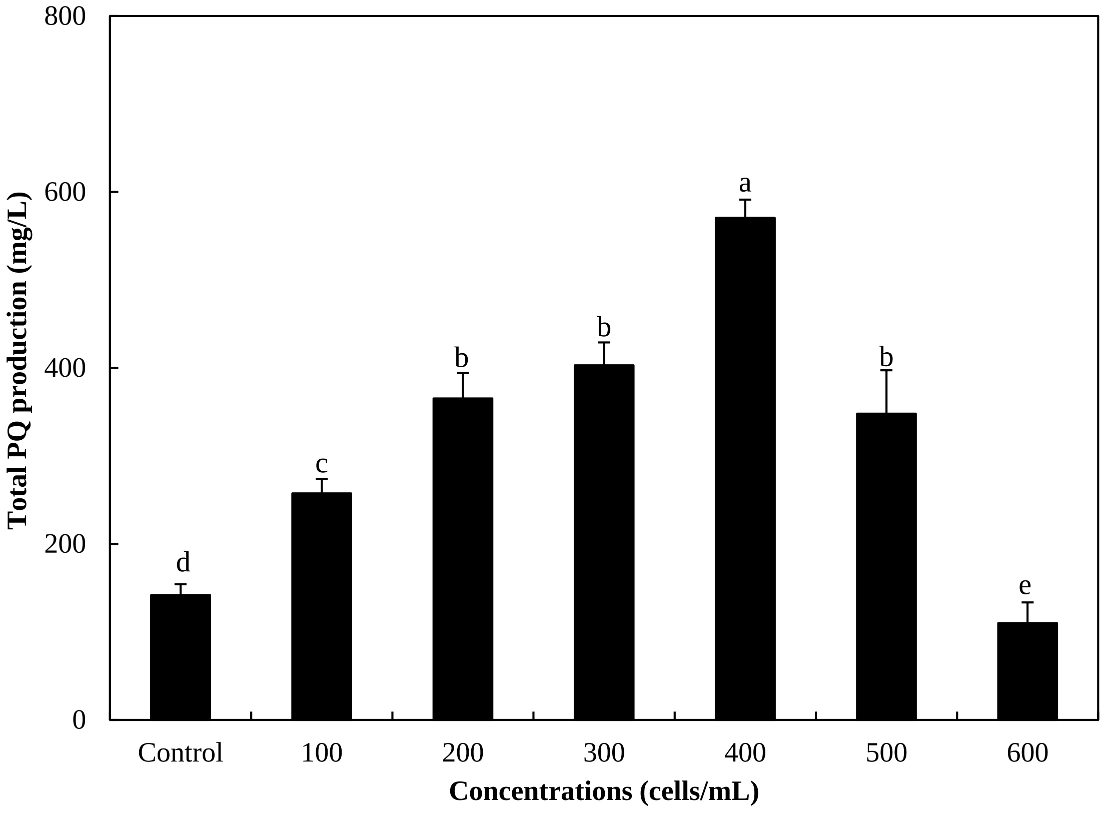

Fig. S1 Effect of inoculum concentration of *Pseudomonas fulva* SB1 on total PQ production in submerged culture of *Shiraia* sp. S9. *P. fulva* SB1 was inoculated on day 6 of mycelium culture at 150 rpm, 28℃. Total PQ production refers to the sum of the intracellular and extracellular PQs. Different *letters* above the bars mean significant differences ($p < 0.05$).

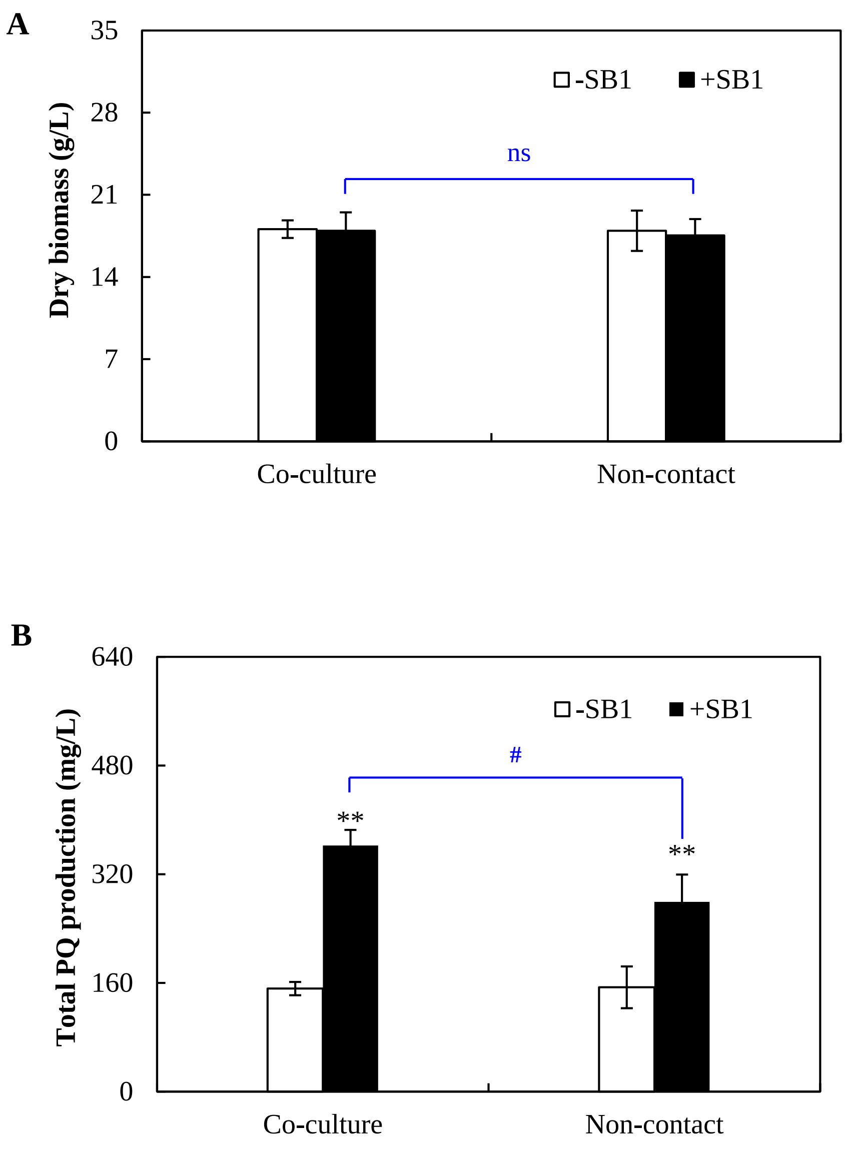

Fig. S2 Effects of *Pseudomonas fulva* SB1 on (**A**) mycelium dry biomass and (**B**) total PQ production in submerged culture of *Shiraia* sp. S9 on day 8. *P. fulva* SB1 was inoculated at 400 cells/mL on day 6 of mycelium culture at 150 rpm, 28°C. Statistical comparison between the two groups showed no significance denoted as "ns". $^{**}p < 0.01$ versus "-SB1" group. "-SB1" group refers control group without the bacteria. $^{\#}p < 0.01$ denotes the significant difference between the "+SB" in fungal co-culture and non-contact co-culture with bacteria.

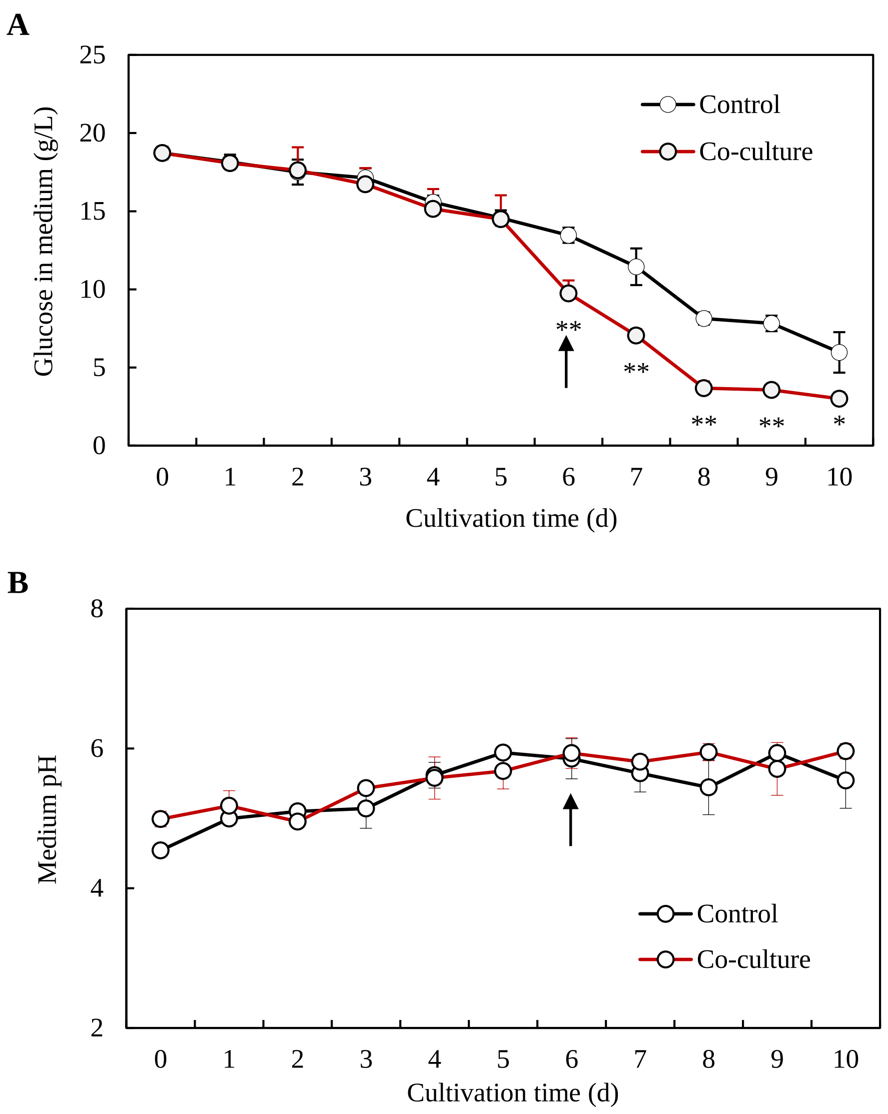

Fig. S3 Time profiles of (**A**) residual sugar and (**B**) medium pH in submerged cultures of *Shiraia* sp. S9 with the addition of *Pseudomonas fulva* SB1. The procedure of co-culture was the same as specified in Figure 3. The *arrow* represents addition time of *P. fulva* SB1. $*p < 0.05$, $**p < 0.01$ versus control group.

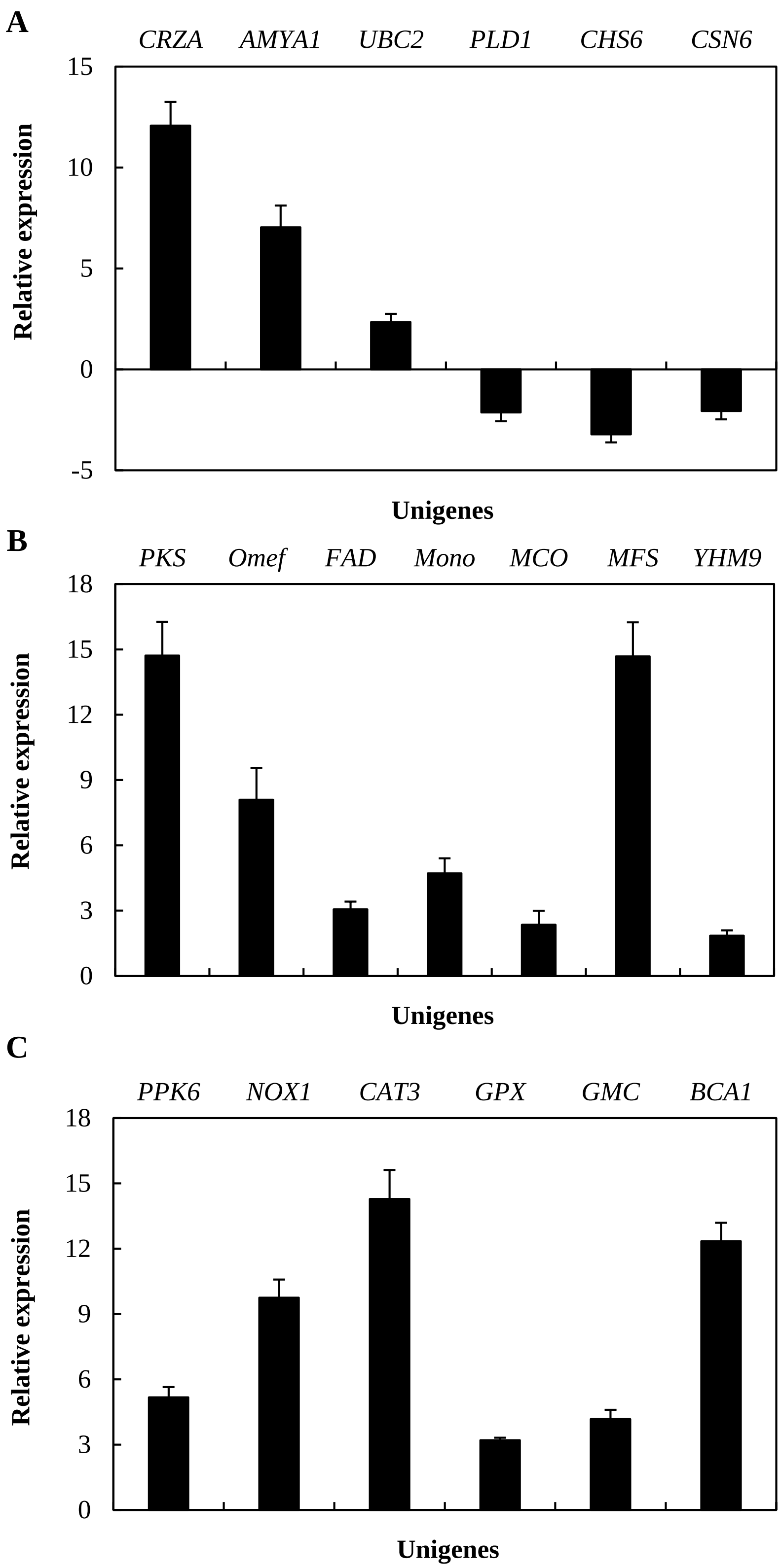

Fig. S4 Validation of the expression levels of genes related to (**A**) growth and development, (**B**) biosynthesis and transport of hypocrellins and (**C**) oxidoreductase activities of co-culture between *Pseudomona fulva* SB1 and *Shiraia* sp. S9 by qPCR. The cultural condition is the same as specified in Fig. 3.

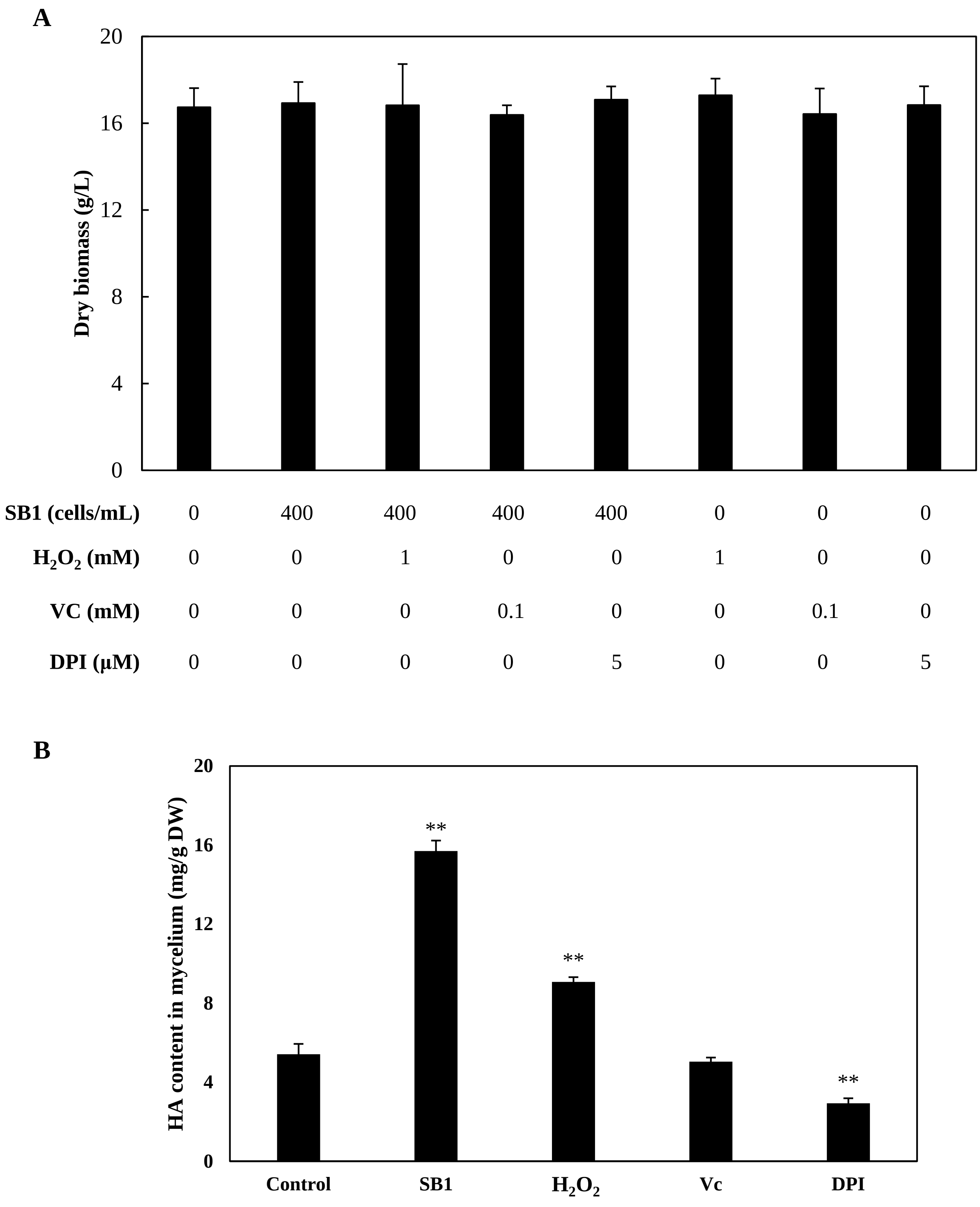

Fig. S5 ROS-dependent modulation of biomass (**A**) and HA biosynthesis (**B**) in *Shiraia* sp. S9 during co-culture with *Pseudomonas fulva* SB1. The optimized co-culture protocol is the same as shown in Fig. 3. H_2O_2, VC and DPI were added 1 h prior to SB1 treatment respectively. ** $p < 0.01$ versus control group without any treatments.
